# Pharmacological Inhibition of SP1 Reverses Cancer Stemness and Enhances Sorafenib Efficacy in Hepatocellular Carcinoma

**DOI:** 10.3390/cells15110961

**Published:** 2026-05-22

**Authors:** Maël Padelli, Christophe Desterke, Aurore Devocelle, Denis Clay, Agnès Bourillon, Georges Uzan, Antoinette Lemoine, Julien Giron-Michel

**Affiliations:** 1 INSERM UMR-S 1193, Université Paris-Saclay, 94800 Villejuif, France; aurore.devocelle@inserm.fr (A.D.); georges.uzan@inserm.fr (G.U.); antoinette.lemoine@aphp.fr (A.L.); julien.giron-michel@inserm.fr (J.G.-M.); 2 Department of Biochemistry and Oncogenetics, Paul Brousse Hospital, AP-HP, 94800 Villejuif, France; agnes.bourillon@aphp.fr; 3 INSERM UMR1310, Université Paris-Saclay, 94800 Villejuif, France; christophe.desterke@inserm.fr; 4 INSERM UMS-44, Hôpital Paul Brousse, Université Paris-Saclay, 94807 Villejuif, France; denis.clay@inserm.fr

**Keywords:** hepatocellular carcinoma, cancer stem cells, drug resistance, sorafenib, specific protein 1, mithramycin A, epigenetics

## Abstract

**Highlights:**

**What are the main findings?**

- SP1 is overexpressed in HCC and independently associated with shorter disease-free survival.
- Mithramycin A (MIT-A) suppresses stemness-associated transcriptional programs and CSC phenotypes in HCC cells and restores sorafenib sensitivity.

**What is the implication of the main finding?**

- SP1-associated GC-rich transcriptional networks represent a potential therapeutic vulnerability in sorafenib-resistant HCC.
- MIT-A exerts broader GC-rich transcriptional and epigenetic effects than isolated SP1 depletion, likely involving additional SP-family transcription factors.

**Abstract:**

Hepatocellular carcinoma (HCC) is a highly heterogeneous malignancy characterized by poor prognosis and limited therapeutic response. Cancer stem cells (CSCs) contribute to tumor progression, therapeutic resistance, and tumor recurrence. Among transcriptional regulators potentially involved in these processes, Specificity Protein 1 (SP1) has emerged as a candidate integrator of oncogenic and epigenetic signaling networks. However, its contribution to CSC-associated phenotypes and drug resistance in HCC remains incompletely defined. In this study, we combined transcriptomic analyses of TCGA datasets with functional experiments in HCC cell lines (Huh7 and HepG2). SP1-associated transcriptional programs were targeted pharmacologically using mithramycin A (MIT-A) and genetically using siRNA-mediated knockdown. The effects were assessed by RNA sequencing, RT-qPCR, Western blotting, flow cytometry, and functional assays evaluating proliferation, migration, CSC-associated properties, and response to sorafenib. MIT-A treatment markedly reduced the expression of stemness-associated transcription factors (NANOG, OCT4, SOX2) and CSC markers (CD133, CD24), impaired CSC-related functions including ALDH activity and the Side Population phenotype, and inhibited cell proliferation and migration. MIT-A also sensitized both parental and sorafenib-resistant HCC cells to sorafenib, associated with modulation of apoptotic regulators and reduced transporter-mediated efflux activity. SP1 knockdown partially reproduced several of these effects, supporting a contribution of SP1-dependent transcriptional programs to these phenotypes. Overall, these findings identify SP1-associated transcriptional networks as potential regulators of CSC features and therapeutic resistance in HCC and support targeting SP1-associated transcriptional programs as a strategy to enhance sorafenib efficacy.

## 1. Introduction

Hepatocellular carcinoma (HCC) is the most common primary liver cancer and the fourth leading cause of cancer-related mortality worldwide [1]. Despite advances in surveillance, molecular classification, and systemic therapies, prognosis remains poor due to the high intra-tumoral heterogeneity of HCC, the persistence of cancer stem cell (CSC) populations, and the development of resistance to targeted therapies and immunotherapy [2]. These CSC resistance mechanisms encompass dysregulation of ABC membrane transporters, induction of cell cycle arrest leading to quiescent states, enhanced DNA repair efficiency, and heightened resistance to anticancer drug-induced apoptosis [3]. Moreover, CSCs have been implicated in tumor initiation, metastasis, and relapse, as well as in chemoresistance through their ability to self-renew, adapt to microenvironmental stress, and evade cell death. Importantly, CSCs display remarkable plasticity, allowing dynamic interconversion between stem-like and differentiated states depending on microenvironmental cues [4]. The tumor microenvironment (TME), including hypoxia, inflammatory signaling, stromal interactions, and immune evasion mechanisms, acts as a supportive niche that sustains CSCs and fosters therapy resistance [5].

Within this context, transcription factors emerge as central regulators that integrate intrinsic signaling with extrinsic microenvironmental cues to maintain CSC phenotypes. Among them, Specificity Protein 1 (SP1), a ubiquitously expressed GC-box binding transcription factor, has attracted particular attention because of its ability to integrate multiple oncogenic inputs and orchestrate large gene networks involved in proliferation, apoptosis, epithelial–mesenchymal transition (EMT), angiogenesis, and drug resistance [6,7,8]. SP1 also influences gene expression through non-canonical mechanisms, including interaction with chromatin modifiers, lncRNAs that facilitate their recruitment to specific promoters, and RNA or RNA-binding proteins for post-transcriptional control [9,10]. SP1 activity is further tuned by post-translational modifications (phosphorylation, acetylation, SUMOylation, ubiquitination and more), allowing dynamic responses to signaling and stress [7].

SP1 is frequently overexpressed in many solid tumors and has been associated with lymph node metastasis, advanced tumor stage, and reduced survival across cancer types [6]. In diverse malignancies, SP1 has been shown to regulate oncogenic signaling such as MAPK/ERK, PI3K/AKT, and Wnt/β-catenin, thereby contributing to proliferation, tumor angiogenesis, immune evasion, and invasive behavior [11,12,13]. Beyond these canonical oncogenic functions, accumulating evidence across solid tumors supports a role for SP1 in maintaining cancer stem-like phenotypes through transcriptional regulation of core pluripotency factors such as OCT4, NANOG, and SOX2, as well as drug resistance–associated genes including ABC transporters and anti-apoptotic regulators [14,15,16]. In HCC, SP1 overexpression has been consistently reported in tumor tissues and cell lines (e.g., Huh7, Hep3B), where it promotes cell proliferation [17], tumor angiogenesis through the SP1/miR-130b-3p/HOXA5 axis [18], immune evasion via disruption of MerTK signaling [19], and EMT-associated invasiveness through modulation of TGF-β/Smad signaling [20]. Importantly, the SP1-NANOG pathway has been shown to contribute to CSC-like properties in HCC [21].

Despite these observations, the broader role of SP1 in orchestrating CSC-associated transcriptional programs and therapeutic resistance in HCC remains insufficiently characterized. In this study, we systematically investigated the functional roles of SP1 by integrating public transcriptomic data with in vitro assays and pharmacological inhibition strategies. Our findings support a significant contribution of the transcription factor SP1 to liver cancer stemness and drug resistance. We show that pharmacological inhibition of SP1 with mithramycin A (MIT-A) suppresses stemness-associated transcriptional programs, reduces functional CSC properties and migratory capacity, and re-sensitizes both naïve and sorafenib-resistant HCC cells to sorafenib. Complementary SP1 silencing experiments partially recapitulated these effects, notably reducing proliferation, downregulating selected pluripotency-associated factors, and enhancing sorafenib sensitivity. Collectively, our results position SP1 as an important transcriptional node linking stemness-associated transcriptional regulation and therapeutic resistance in HCC, while also suggesting that broader GC-binding-dependent mechanisms may contribute to the full pharmacological phenotype observed with MIT-A.

## 2. Materials & Methods

### 2.1. Cell Culture, Hypoxia Exposure, Drug Treatment and siRNA Transfection

The human HCC cell lines Huh7 (WT) and HepG2 (WT) were obtained from ATCC. Sorafenib-resistant cell lines Huh7 SR and HepG2 SR were generated in our laboratory by continuous culture under gradually increasing concentrations of sorafenib over a two-month period, reaching 10 µM. Cells were cultured in Dulbecco’s Modified Eagle’s Medium (DMEM; Gibco, Thermo Fisher Scientific, Waltham, MA, USA) supplemented with 10% fetal bovine serum (FBS; Gibco), 1% penicillin–streptomycin, at 37 °C in a humidified incubator containing 5% CO_2_. For hypoxia experiments, cells were incubated in a hypoxia workstation (Baker Ruskinn SCI-tive, Bridgend, UK) under 1% O_2_, 94% N_2_, and 5% CO_2_ for 24 h to 48 h. Normoxic controls were maintained at 21% O_2_. Huh7 WT and Huh7 SR cells were treated with the SP1 inhibitor MIT-A (Merck, Darmstadt, Germany, M6891; 200 nM and 400 nM, respectively) for 24 h for RNA sequencing and quantitative PCR, and for 48 h for complementary phenotypic and functional assays. For genetic silencing experiments, cells were transiently transfected with SP1 siRNA (siSP1; Thermo Fisher Scientific, Waltham, MA, USA, #HSS186048, 120–200 pmol per 6 cm dish) using Lipofectamine RNAiMAX Transfection Reagent (Thermo Fisher Scientific, 13778150) according to the manufacturer’s instructions. Analyses were performed 48 h post-transfection unless otherwise specified.

### 2.2. RNA-seq and Transcriptomic Analyses

For host transcriptome profiling, Huh7 WT and sorafenib-resistant cells treated with MIT-A or control were harvested for RNA sequencing. Total RNA was extracted using the Qiagen RNeasy kit (Qiagen, Hilden, Germany, #74104), and RNA integrity was assessed by Nanodrop spectrophotometry and agarose gel electrophoresis. Next generation sequencing (NGS) libraries were prepared using RNA Depletion and RNA Library Preparation with Twist UMI Adapter System kit (Twist Bioscience, South San Francisco, CA, USA) following the manufacturer’s instructions, using 100 ng of RNA as input. Libraries were verified on a D1000 screentape assay using the 4200 TapeStation (Agilent Technologies, Santa Clara, CA, USA). Sequencing was performed on a Nextseq1000 Illumina system (Illumina, Inc., San Diego, CA, USA) by multiplexing 8 samples per flow cell and performing 100 bp paired-end reads.

Bulk RNA-sequencing data for liver hepatocellular carcinoma (LIHC) were retrieved from The Cancer Genome Atlas (TCGA) program. Normalized mRNA expression profiles (RNA-Seq by Expectation Maximization, RSEM) and clinical annotations were downloaded from the Genomic Data Commons (GDC) Firehose pipeline through cBioPortal (https://www.cbioportal.org/, accessed 13 February 2023). Only primary tumor samples with complete transcriptomic and clinical information were *included* (*n* = 371, Supplementary Table S1). For survival analyses, only patients with available disease-free survival (DFS) data were retained, resulting in a subset of 319 patients. Patients were stratified according to SP1 expression Z-scores (±1.96 SD). For clinical correlation and stratification analyses, RSEM-normalized values were directly used. For analyses requiring raw count data, count matrices were normalized using the edgeR Bioconductor package (v3.38.4) to account for library size and composition bias. Clinical and expression data were integrated with the dplyr R package (v1.0.10). Gene Set Enrichment Analysis (GSEA v4.0.3) was performed using the MSigDB database (v6.2), including Hallmark gene sets. Visualization was achieved using ggplot2 (boxplots), FactoMineR (PCA), and pheatmap (heatmaps). Differential expression was considered significant at FDR < 0.05. Stemness features were quantified using CancerStemnessOnline (http://bio-bigdata.hrbmu.edu.cn/CancerStemnessOnline, accessed on 7 November 2023), focusing on the Stemness Index, where higher values indicate a more progenitor-like phenotype [22], and by analyzing the curated collection of cancer stemness gene sets available on this platform [23,24].

### 2.3. RNA Extraction and Quantitative RT-PCR

Total RNA was extracted using the Qiagen RNeasy kit (Qiagen, Hilden, Germany, #74104), and RNA integrity was assessed by Nanodrop spectrophotometry. Complementary DNA (cDNA) was synthesized from 500 ng of total RNA using SuperScript III One-Step RT-PCR System with Platinum Taq High Fidelity (Thermo Fisher Scientific, Waltham, MA, USA, #12574035). Quantitative PCR was performed with TaqMan Universal PCR Master Mix (Thermo Fisher Scientific, Waltham, MA, USA, #4318157) on a StepOnePlus system (Applied Biosystems, Thermo Fisher Scientific, Waltham, MA, USA). 18S and GAPDH served as endogenous controls. Relative expression was calculated using the delta-delta CT method. Primer sequences are provided in Supplementary Table S2. To ensure the authenticity of the results, the experiments were performed in at least triplicate.

### 2.4. Flow Cytometry Analyses

#### 2.4.1. CSC Surface Markers

Cells were detached with Accutase (Sigma-Aldrich, Merck, Darmstadt, Germany, #A6964), washed, and resuspended in staining buffer (PBS with 0.5% BSA). For each condition, 1 × 10^5^ cells were incubated with Fluorescein isothiocyanate (FITC)-, phycoerythrin (PE)- or APC-conjugated antibodies targeting CD24 (Immunotools, Friesoythe, Germany, #21270244 × 2), CD29 (Immunotools, Friesoythe, Germany, #21270294), CD44 (Immunotools, Friesoythe, Germany, #21270444), CD73 (Thermo Fisher Scientific, Waltham, MA, USA, #12-0739-42), CD90 (Thermo Fisher Scientific, Waltham, MA, USA, #12-0909-42), CD105 (Immunotools, Friesoythe, Germany, #21271056), CD133 (Thermo Fisher Scientific, Waltham, MA, USA, #17-1338-42) and EpCAM (Thermo Fisher Scientific, Waltham, MA, USA, #MA5-46684), or with appropriate isotype controls. Acquisition was performed on a BD LSR Fortessa™ cytometer (BD Biosciences, Ashland, OR, USA), and data were analyzed with FlowJo software (FlowJo v10.10, BD Biosciences, Ashland, OR, USA). At least 10,000 events were collected per sample. The experiment was repeated at least three times.

#### 2.4.2. Aldefluor Assay (ALDH Activity)

CSC-associated aldehyde dehydrogenase (ALDH) activity was measured using the Aldefluor kit (StemCell Technologies, Vancouver, BC, Canada, #01700) according to manufacturer’s instructions. Cells were incubated with the ALDH substrate (BAAA) for 45 min at 37 °C with or without the ALDH inhibitor DEAB (10 μM). Cells were then analyzed by flow cytometry (BD Biosciences, San Jose, CA, USA).

#### 2.4.3. Side Population (SP) Assay

The SP fraction was identified using the Hoechst 33,342 dye efflux assay [25]. Briefly, 1 × 10^6^ cells/mL were suspended in prewarmed phenol-free DMEM (Sigma-Aldrich, Merck, Darmstadt, Germany) with 2% FBS. Hoechst 33,342 dye (bisBenzimide HOE 33342; Sigma-Aldrich, Merck, Darmstadt, Germany, #B2261) was added to a final concentration of 5 μg/mL in the presence or absence of 50 µM of verapamil (Sigma-Aldrich, Merck, Darmstadt, Germany). Cells were incubated at 37 °C for 90 min, with intermittent shaking. After washing with phenol-free DMEM, cells were resuspended in ice-cold PBS with propidium iodide (PI) for dead cell exclusion and analyzed by dual-wavelength flow cytometry (blue: 402–446 nm, red: 650–670 nm).

#### 2.4.4. ABC Transporter Efflux Assays

To assess drug efflux activity, cells were incubated in pre-warmed DMEM + 2% FBS containing fluorescent substrates specific for ABC transporters: purpurin-18 (ABCG2 substrate) [26], DIOC2(3) (ABCB1 substrate), or CMFDA (ABCC transporter substrate), in the presence or absence of selective inhibitors: Ko143 (ABCG2 inhibitor, 1 μM; Tocris, Bio-Techne, Bristol, UK, #3241), CP100-356 hydrochloride (ABCB1 inhibitor, 1 μM; Tocris, Bio-Techne, Bristol, UK, #4193/10), or probenecid (ABCC inhibitor, 10 μM; Tocris, Bio-Techne, Bristol, UK, #4107/50). After 30 min of substrate loading at 37 °C, cells were washed to remove extracellular dye and resuspended in pre-warmed DMEM, with or without the corresponding inhibitor. After an additional 1 h incubation at 37 °C to allow efflux, dye retention reflecting intracellular accumulation was quantified by flow cytometry based on the fluorescence intensity of each substrate.

### 2.5. Immunofluorescence

Cells were seeded on Lab-Tek chamber slides Lab-Tek, (Thermo Fisher Scientific, Waltham, MA, USA) (2 × 10^5^ cells/well) and when the cells reached 80% confluence, cells were treated with MIT-A at indicated concentrations and durations. Cells were then fixed in 4% paraformaldehyde, and permeabilized with 0.5% Triton X-100. After blocking with 10% goat serum, slides were incubated with primary antibodies against SP1 (Santa Cruz Biotechnology, Dallas, TX, USA, #sc-420, 1/200), SETDB1 (Thermo Fisher Scientific, Waltham, MA, USA, #MA5-15722, 1/500), followed by Alexa Fluor 488/594-conjugated secondary antibodies. F-actin organization was revealed by staining the cells with 0.2 μg/mL of rhodamine-conjugated phalloidin (Thermo Fisher Scientific, Waltham, MA, USA, #R415) for 20 min. The cells were washed twice with PBS 1X, the nuclei were counterstained with DAPI. Coverslips were mounted in DABCO-glycerol (Thermo Fisher Scientific, Waltham, MA, USA, #P36934) and imaged using a fluorescence microscope (Leica Microsystems, Wetzlar, Germany).

### 2.6. Western Blotting

Total protein extracts were prepared in RIPA buffer supplemented with protease inhibitors. Equal amounts (40 μg) were separated by SDS-PAGE, transferred onto PVDF membranes, and blocked in 5% milk/TBST. Membranes were probed with primary antibodies against SP1 (Santa Cruz Biotechnology, Dallas, TX, USA, #sc-420, 1/200), SETDB1 (Abcam, Cambridge, UK, #ab-107225, 1/2000), XIAP (BD Transduction Laboratories, BD Biosciences, San Jose, CA, USA, #Mab 59520, 1/500), BCL-XL (Santa Cruz Biotechnology, Dallas, TX, USA, #sc634, 1/200), BAX (NT) (Upstate, Merck Millipore, Burlington, MA, USA, #06-499, 1/250), E-cadherin (BD Biosciences, San Jose, CA, USA, #610181, 1/1000), EpCam (Bio-Techne, Minneapolis, MN, USA, #AF960, 1/1000), vimentin (Cell signaling Technology, #73260, 1/1000), SOX2 (Bio-Techne, Minneapolis, MN, USA, #MAB2018, 1/500), Oct4 (Bio-Techne, Minneapolis, MN, USA, #AF1759, 1/1000), Nanog (Santa Cruz Biotechnology, Dallas, TX, USA, #sc-134218, 1/200), CD133 (Miltenyi Biotec, Bergisch Gladbach, Germany, #130-092-395, 1/200), CD24 (Santa Cruz Biotechnology, Dallas, TX, USA, #sc11406, 1/200), GAPDH (Santa Cruz Biotechnology, Dallas, TX, USA, #sc-47724, 1/500) and β-actin (Cell Signaling Technology, Danvers, MA, USA, #47778, 1/15,000), followed by HRP-conjugated secondary antibodies. Signal was revealed with ECL substrate and quantified using ImageJ v1.54m software (NIH, Bethesda, MD, USA).

### 2.7. Cell Proliferation and Migration Assays

#### 2.7.1. Proliferation

Cell viability assay. Cell viability was assessed using the CellTiter-Glo^®^ Luminescent Assay (Promega Corporation, Madison, WI, USA). Cells were seeded in 96-well plates at a density of 3000 cells per well. After 24 h, cells were treated with or without MIT-A (100–200 nM) for 24 h, and then washed and further cultured in complete DMEM medium for an additional 72 h. Luminescence was measured using a microplate reader (Bio-Rad Laboratories, Hercules, CA, USA) at different time points from 24 h to 72 h.

#### 2.7.2. Scratch Wound Healing Assay

Cells were seeded in 6-well plates to confluence, serum-starved with mitomycin C (Merck, #M4287, 10 μg/mL) for 2 h, and scratched with a sterile 200 μL pipette tip. Detached cells were washed away, and wound closure was monitored for up to 72 h under a phase-contrast microscope to calculate the rate of wound closure. Images were analyzed with Image-J v1.54m software (NIH, Bethesda, MD, USA).

### 2.8. Drug Sensitivity Assay and Apoptosis Assays

Cells were seeded in quadruplicate at 3000 cells per well in 96-well plates and incubated overnight at 37 °C in a humidified environment containing 5% CO_2_. Then, increasing concentrations of sorafenib (0 to 50 μM) were added to wells and cultures were incubated for an additional 48 h. Cell viability was determined using the CellTiter-Glo luminescent cell viability kit from Promega Corporation (Promega Corporation, Madison, WI, USA) according to the manufacturer’s instructions. Luminescence was read using a microplate reader (Bio-Rad Laboratories, Hercules, CA, USA). IC_50_ values were calculated by nonlinear regression using GraphPad PRISM v10.4.2 software (GraphPad, San Diego, CA, USA) and are presented as mean ± SEM of relative luminescence values from three independent experiments. Cell viability was also assessed using fluorescein diacetate (FDA) staining. After treatment with sorafenib ± MIT-A, cells were incubated for 5 min with FDA (ThermoFisher Scientific #F1303, 0.2 mg/mL) at 37 °C. Viable cells metabolized FDA into a fluorescent signal, which was quantified by flow cytometry.

### 2.9. Statistical Analysis

All experiments were performed using at least three independent biological replicates unless otherwise stated. Quantitative data are presented as mean ± standard error of the mean (SEM). Statistical analyses were performed using GraphPad Prism v10.4.2 software (GraphPad, San Diego, CA, USA) and R (version 4.4.2). Normality and homogeneity of variance were assessed prior to applying parametric tests. For comparisons between two groups, Student’s *t*-test or Welch’s *t*-test was used as appropriate. For comparisons involving more than two groups or multiple conditions, one-way or two-way ANOVA was applied, followed by appropriate post hoc tests.

For RNA-sequencing analyses, differential gene expression was performed using the edgeR Bioconductor package, with normalization to account for library size and composition bias. Statistical significance for transcriptomic analyses was determined using false discovery rate (FDR) correction (Benjamini–Hochberg method), with FDR < 0.05 considered significant.

The specific statistical tests used for each experiment are indicated in the corresponding figure legends. A *p* value < 0.05 was considered statistically significant.

## 3. Results

### 3.1. SP1 Is Overexpressed in Hepatocellular Carcinoma and Predicts Poor Prognosis

**Figure 1:**
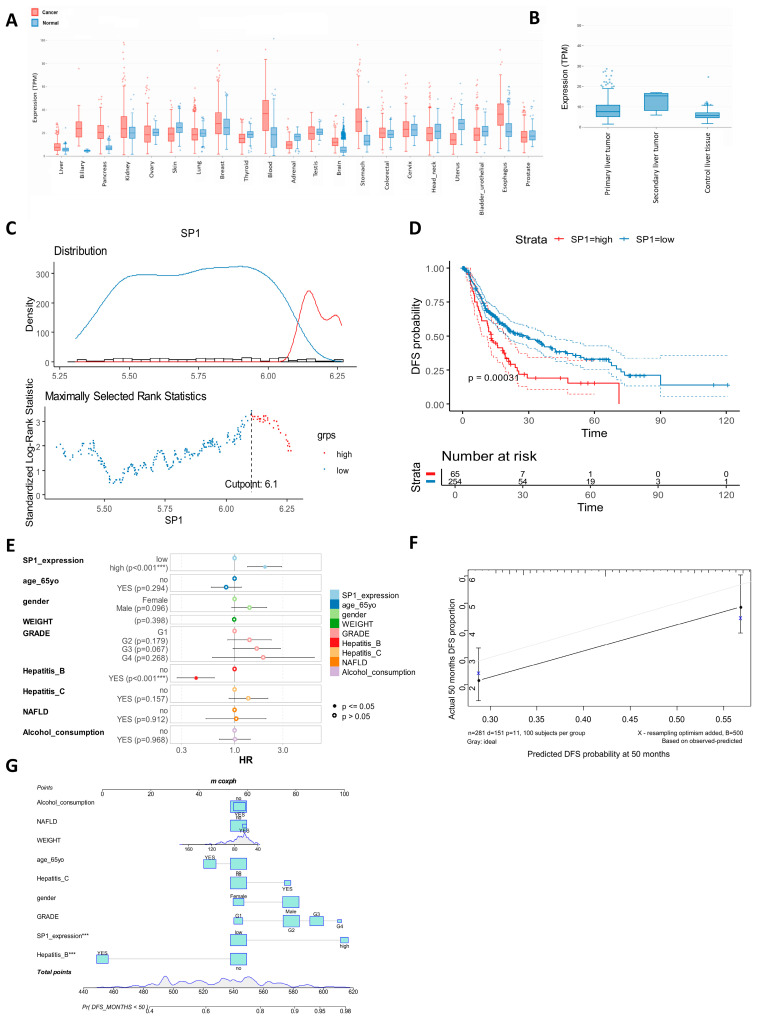
Analyses of SP1 expression and prognostic value in HCC. (**A**) Pan-cancer analysis of SP1 mRNA expression using TCGA datasets, represented as boxplots across multiple tumor types. (**B**) Comparison of SP1 expression between primary HCC, secondary liver tumors, and non-tumoral liver tissues, represented as boxplots. (**C**) Determination of the optimal cutoff for SP1 expression using the maximum rank statistic (maxstat R package). The panel shows the distribution of SP1 expression values with the defined threshold. (**D**) Kaplan–Meier disease-free survival (DFS) analysis stratified by SP1 expression levels **(red: SP1-high group; blue: SP1-low group; dashed lines: 95% confidence intervals)**. (**E**) Forest plot from multivariate Cox regression analysis for DFS **(filled circles: *p* ≤ 0.05; open circles: *p* > 0.05)**. In panels (**A**,**B**), **red boxes represent tumor/cancer samples and blue boxes represent normal/control tissues**. (**F**) Predicted 50-month DFS probability according to SP1 expression categories based on the multivariable Cox model. (**G**) Association of SP1 expression with clinical and histopathological features (tumor grade, stage, vascular invasion, etc.), represented as heatmaps. Statistical analyses included Student’s *t*-test or χ^2^ test for group comparisons, the log-rank test for Kaplan–Meier survival analyses, and Cox proportional hazards regression for multivariable survival analyses (*n* = 319 for survival analyses; total cohort *n* = 371; α = 0.05).

To investigate the clinical significance of SP1, we first analyzed its expression across TCGA pan-cancer datasets. SP1 was significantly upregulated in several tumor types compared with normal tissues, including HCC (Figure 1A). Within the liver cohort, SP1 expression was markedly elevated in primary HCC tumors compared with non-tumoral liver tissue (*p* < 0.001) (Figure 1B). Interestingly, secondary liver tumors (metastatic lesions) exhibited even higher SP1 expression than primary HCCs, highlighting a potential role of SP1 in aggressive and metastatic disease biology (Figure 1B).

The TCGA-LIHC cohort included heterogeneous etiologies of underlying liver disease, including HBV (29.5%), HCV (15.9%), alcohol-related liver disease (33.2%), and NAFLD-associated cases (5.7%) (Supplementary Table S1). Exploratory analyses revealed no major differences in SP1 expression according to HBV, HCV, or NAFLD status within the TCGA-LIHC cohort (Supplementary Figure S1). Although a marginal statistical difference was observed between HCV-associated and non-HCV tumors, the magnitude of this variation was minimal. These findings suggest that SP1 overexpression is broadly conserved across etiological HCC subtypes rather than being restricted to a specific viral or metabolic background.

Using a maximally selected rank statistic approach, we determined the optimal cutoff for SP1 expression (≥6.1 for normalized expression) that best separated patients into high (*n* = 65) and low (*n* = 254) groups (Figure 1C). This cutoff was derived in a data-driven manner and should therefore be interpreted as exploratory, as it was not validated in an independent cohort. Kaplan–Meier analysis of disease-free survival (DFS) revealed that patients in the SP1-high group experienced significantly shorter DFS than those in the SP1-low group (log-rank *p* = 1 × 10^−4^; Figure 1D), indicating an association between high SP1 expression and increased risk of disease recurrence.

To assess whether SP1 expression retained prognostic value independently of established clinicopathological variables, a multivariable Cox proportional hazards model was constructed including age, sex, tumor grade, and etiology. In this multivariable DFS analysis, high SP1 expression remained an independent predictor of poor prognosis (HR = 1.98, 95% CI = 1.34–2.91, *p* < 0.0001; Figure 1E). The model showed a moderate discriminative performance, with a concordance index (C-index) of 0.634 ± 0.025. The proportional hazards assumption of the Cox model was assessed using Schoenfeld residuals and was not violated (global test *p* = 0.07). The multivariable DFS model was further calibrated using 500 bootstrap resampling iterations to estimate predicted disease-free survival probability at 50 months (Figure 1F). The resulting nomogram highlighted SP1 expression as a major contributor to the overall prognostic score relative to other clinical variables (Figure 1G). Together, these results support a role for SP1 as an oncogenic-associated factor in HCC, whose overexpression is linked to earlier disease recurrence and independently associated with poor disease-free survival.

### 3.2. SP1 Expression Correlates with Stemness Markers in Hepatocellular Carcinoma

**Figure 2:**
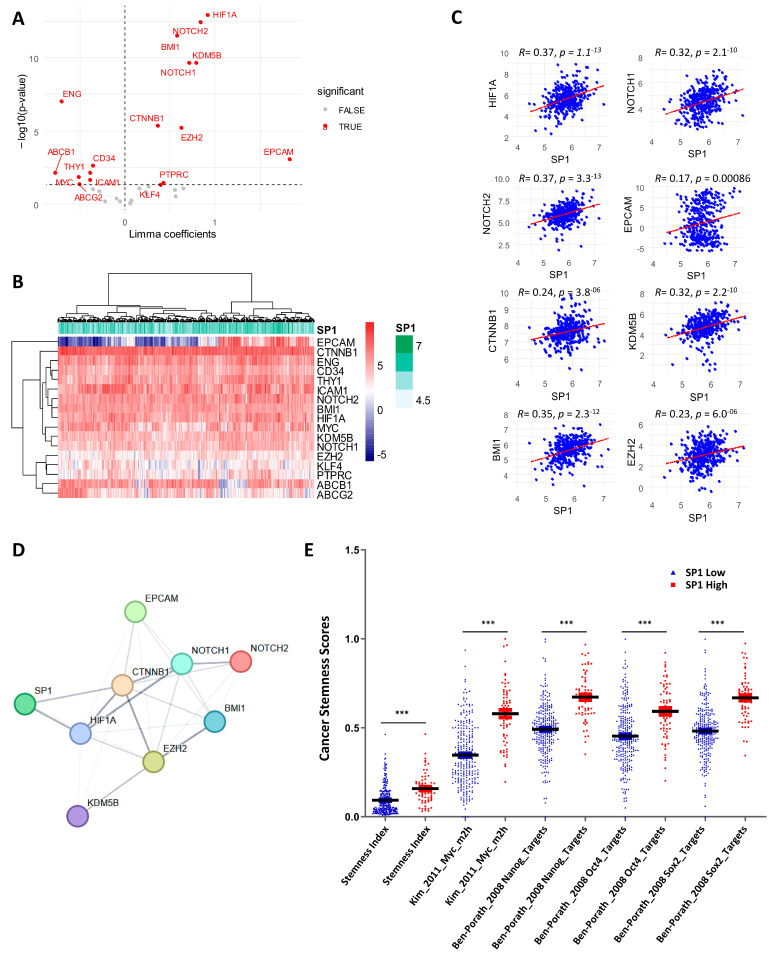
Correlation between SP1 expression and stemness-associated transcriptional programs. (**A**) Correlation plot displaying the association between SP1 expression and all genes in the TCGA-LIHC cohort. The *x*-axis represents the limma regression coefficient (effect size) and the *y*-axis –log10(*p*-value). Stemness-associated regulators are highlighted. (**B**) Heatmap of the 17 stemness-related genes most significantly associated with SP1 (adjusted *p* < 0.05). Each column represents an individual patient and each row a gene, revealing a coherent clustering of SP1 with canonical stemness regulators. (**C**) Scatter plots showing Pearson correlations between SP1 expression and representative stemness-associated genes (*HIF1A*, *NOTCH1*, *NOTCH2*, *BMI1*, *EPCAM*, *EZH2*, *CTNNB1*, *KDM5B*). Regression lines, correlation coefficients (r), and *p*-values are indicated. (**D**) Protein–protein interaction (PPI) network generated with the STRING database, highlighting functional connections between SP1 and stemness regulators identified in panel (**A**). (**E**) Functional validation of the association between SP1 and cancer stemness programs. Stemness features were quantified using CancerStemnessOnline. The Stemness Index was compared between SP1-High and SP1-Low groups, and enrichment analyses were performed with canonical stemness gene sets (Kim_et_al_Myc_m2h, Ben_Porath_Myc_targets1, Ben_Porath_Nanog, Oct4, Sox2, c-Myc). Statistical significance was assessed by Welch’s *t*-test (*** *p* < 0.001).

Given the prognostic relevance of SP1, we next investigated its association with stemness-related transcriptional programs in HCC using transcriptomic data from the TCGA-LIHC cohort. Transcriptomic analyses first revealed that SP1 expression was significantly correlated with a panel of stemness-associated genes (Figure 2A). A focused heatmap including the 17 genes most significantly associated with SP1 (adjusted *p* < 0.05) demonstrated a coherent clustering pattern, with SP1 grouping alongside canonical stemness regulators such as *HIF1A*, *NOTCH1*, *NOTCH2*, *BMI1*, *EPCAM*, *EZH2*, *CTNNB1*, and *KDM5B* (Figure 2B). Correlation analyses further confirmed the robustness of these positive associations, with Pearson coefficients ranging from r = 0.17 to r = 0.37 (Figure 2C). In parallel, a protein–protein interaction network built from the SP1-associated gene set highlighted functional connections linking SP1 to multiple stemness regulators (Figure 2D). To further validate these findings, we investigated whether SP1 expression is functionally associated with cancer stemness programs in HCC. Using CancerStemnessOnline [22], we quantified stemness features and found that the Stemness Index was significantly elevated in the SP1-high group, indicating a stronger stem-like transcriptional identity. Moreover, enrichment analyses demonstrated a significant positive correlation between SP1 expression and canonical stemness gene sets, including Kim_et_al_Myc_m2h, Ben_Porath_Nanog, Ben_Porath_Oct4, Ben_Porath_Sox2 (Figure 2E). Taken together, these results indicate that SP1 overexpression is tightly coupled with transcriptional networks governing stemness in HCC, thereby providing a mechanistic link between its prognostic impact and biological function.

### 3.3. Pharmacological Inhibition of SP1 with Mithramycin A Reduces Proliferation and Migration

To investigate the role of SP1 in HCC, we employed three human HCC cell lines: Huh7 (WT), its sorafenib-resistant derivative (Huh7 SR), and HepG2. The Huh7 SR subline was generated in our laboratory by continuous exposure to increasing concentrations of sorafenib over a two-month period, reaching 10 µM. Morphologically, Huh7 SR cells exhibited a spindle-like, mesenchymal phenotype compared to the epithelial-like appearance of Huh7 WT cells, as observed by phase-contrast microscopy (Supplementary Figure S2A). At the molecular level, Western blotting confirmed reduced expression of the epithelial marker E-cadherin and increased expression of the mesenchymal marker vimentin in Huh7 SR compared with Huh7 WT, consistent with an EMT phenotype (Supplementary Figure S2B).

**Figure 3:**
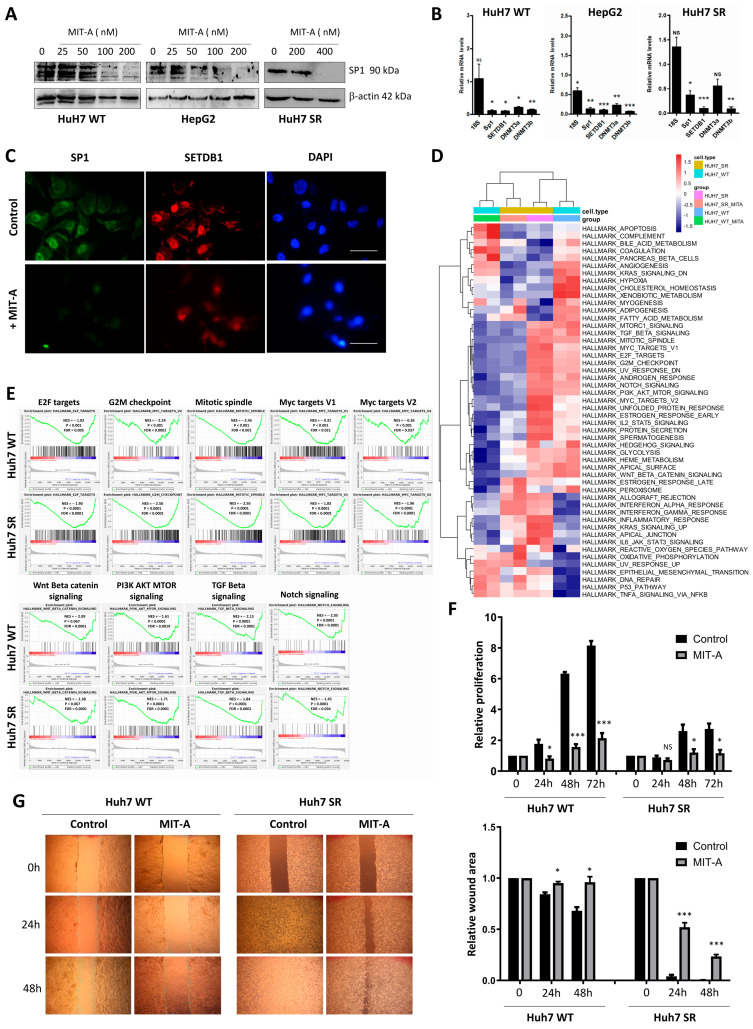
Effect of MIT-A on SP1/SETDB1 axis, proliferation, and migration. (**A**) Western blot analysis of SP1 in Huh7 WT, Huh7 SR, and HepG2 WT cells treated for 48 h with increasing concentrations of Mithramycin-A (MIT-A, 25–400 nM). β-actin served as loading control. (**B**) RT-qPCR analysis of SP1 and SP1 targets (SETDB1, DNMT3A, DNMT3B) in HCC cell lines treated for 24 h with MIT-A (200 nM for Huh7 WT and HepG2; 400 nM for Huh7 SR). Expression was normalized to 18S. (**C**) Immunofluorescence analysis of SP1 and SETDB1 localization in Huh7 WT cells treated with MIT-A (200 nM, 24 h). Representative images show nuclear staining (DAPI, blue) and cytoplasmic/nuclear SP1/SETDB1 staining (green). Scale bars = 50 μm. (**D**) RNA-seq analysis followed by GSEA using the Hallmark gene set collection (MSigDB) in Huh7 WT and SR cells treated with MIT-A. Heatmap of enrichment scores for 50 Hallmark pathways. (**E**) GSEA plots showing enrichment of proliferation- and stemness-related signatures in MIT-A–treated HCC cells compared with untreated controls. Normalized Enrichment Score (NES), *p*-value, and False Discovery Rate (FDR) q-values are indicated. (**F**) Cell proliferation (0–72 h) measured by CellTiter-Glo in Huh7 WT and SR cells pretreated or not with MIT-A (200 nM, 24 h for Huh7 WT; 400 nM, 24 h for Huh7 SR). Values are normalized to the initial time point. (**G**) Scratch wound healing assay of Huh7 WT and SR cells pre-exposed to MIT-A (24 h). Left, representative images of wound closure at 0, 24, and 48 h. Right: quantification of wound closure expressed as percentage of closure. Data are presented as mean ± SEM from independent experiments ((**B**): *n* = 3; (**F**): *n* = 5; (**G**): *n* = 3). Statistical analyses were performed using unpaired two-tailed Student’s *t*-tests for single comparisons (**B**) and two-way ANOVA followed by Sidak’s multiple comparisons test for proliferation and migration assays (**F**,**G**). Statistical significance is indicated as * *p* < 0.05, ** *p* < 0.01, *** *p* < 0.001; NS, not significant.

To functionally interrogate SP1, we subsequently treated all three cell lines with MIT-A, a pharmacological inhibitor of SP1 binding to GC-rich promoter regions. Western blot analyses demonstrated a dose-dependent inhibition of SP1 protein expression by MIT-A across three HCC cell lines (HuH7WT, HepG2, and SR), although the effective dose varied; 200 nM for Huh7WT and HepG2, and 400 nM for SR cells (Figure 3A). To determine whether MIT-A treatment also affected SP1-associated transcriptional programs, we performed RT-qPCR analysis of SP1 and representative SP1-regulated targets, including *SETDB1*, *DNMT3A*, and *DNMT3B*, in HCC cells treated at the effective concentrations identified above. Consistent with the protein-level inhibition, MIT-A significantly reduced SP1 transcript abundance together with a coordinated downregulation of its downstream targets in all three cell lines (Figure 3B). Immunofluorescence analysis further supported these results. In untreated Huh7WT cells, SP1 was detected in both the nucleus and cytoplasm, whereas SETDB1 was predominantly nuclear. MIT-A treatment (200 nM, 48h) induced a strong reduction in SP1 in both compartments and led to loss of nuclear SETDB1 staining, confirming transcriptional and subcellular suppression of the SP1/SETDB1 axis (Figure 3C).

To gain insight into global transcriptional changes, we performed RNA-seq followed by Gene Set Enrichment Analysis (GSEA) across 50 canonical Hallmark pathways (Figure 3D,E). MIT-A treatment led to a broad suppression of proliferation-related programs, including E2F targets, G2M checkpoint, and mitotic spindle, consistent with a loss of proliferative capacity. Furthermore, stemness-associated signaling pathways such as MYC targets v1/v2, WNT/β-catenin, PI3K-AKT-mTOR, TGF-β, and NOTCH were significantly downregulated in MIT-A–treated cells. Together, these data demonstrate that pharmacological interference with GC-rich transcriptional programs disrupts both cell cycle progression and stemness-related transcriptional programs. Functional validation using CellTiter-Glo proliferation assays in Huh7 WT and SR cells confirmed the anti-proliferative effect of MIT-A (200 nM). Compared with untreated controls, MIT-A significantly reduced cell proliferation in a time-dependent manner, with significant effects observed from 24 h onward in Huh7 WT cells and at later time points in Huh7 SR cells (Figure 3F), consistent with cell cycle arrest and impaired growth.

MIT-A also impaired HCC cell motility. In scratch wound assays, MIT-A–treated cells showed markedly reduced wound closure compared with controls, as quantified over time. This effect was particularly pronounced in the Huh7 SR subline, which displays higher basal migratory activity than Huh7WT (Figure 3G). To explore whether this loss of migration involved modulation of EMT, we first examined the expression of key EMT-related genes. RNA-seq analysis showed that in Huh7 WT cells, MIT-A decreased *CDH1* and *ZEB1* while increasing *VIM*, whereas in Huh7 SR cells MIT-A reduced *CDH1*, *ZEB2*, and VIM (Supplementary Figure S2C). Western blot validation of selected markers confirmed these trends: MIT-A decreased E-cadherin and increased vimentin in Huh7 WT, while in Huh7 SR it reduced E-cadherin without affecting vimentin expression (Supplementary Figure S2D). Extending this analysis to pathway-level changes, GSEA revealed that MIT-A treatment yielded a normalized enrichment score (NES) of 2.12 for the HALLMARK EMT signature in Huh7 WT, indicating partial EMT modulation, whereas in Huh7 SR the NES was −0.83, suggesting minimal effect (Supplementary Figure S2E). These results indicate that the loss of migration cannot be fully explained by EMT modulation, as EMT-related changes were heterogeneous and not consistently associated with the migratory phenotype, suggesting the involvement of alternative mechanisms.

Furthermore, RNA-seq analysis revealed that MIT-A markedly downregulated the HGF/c-MET signaling axis, a key upstream driver of motility and invasion in HCC, through reduced expression of MET and multiple downstream adaptors and effectors involved in migration-related signaling, including GRB2, CRK, SOS1, MAPK1/3, MAP2K1, PTK2 (FAK), and PIK3CA. Concomitantly, MIT-A downregulated Rho GTPases and their regulators (e.g., RAC1, RHOA), the Arp2/3 actin remodeling machinery (e.g., ACTR2, ACTR3), as well as integrins and focal adhesion components (e.g., ITGB3, TLN1), indicating a global impairment of cytoskeletal dynamics and adhesion turnover required for migration (Supplementary Figure S2F).

Complementary immunofluorescence experiments confirmed these transcriptomic findings, showing that MIT-A-treated cells lost actin-based protrusions and adopted a more rounded morphology compared with controls (Supplementary Figure S2G). Overall, these results suggest that MIT-A suppresses HCC cell migration through transcriptional modulation of pathways controlling cytoskeleton dynamics and adhesion, rather than through a consistent EMT-associated mechanism.

Collectively, pharmacological interference with GC-rich transcriptional programs by MIT-A reduces SETDB1 expression, silences proliferation- and stemness-related transcriptional programs, and impairs both growth and migration in parental and sorafenib-resistant HCC cells.

### 3.4. Mithramycin A Suppresses Stemness-Associated Markers and Phenotypes in HCC Cells

**Figure 4:**
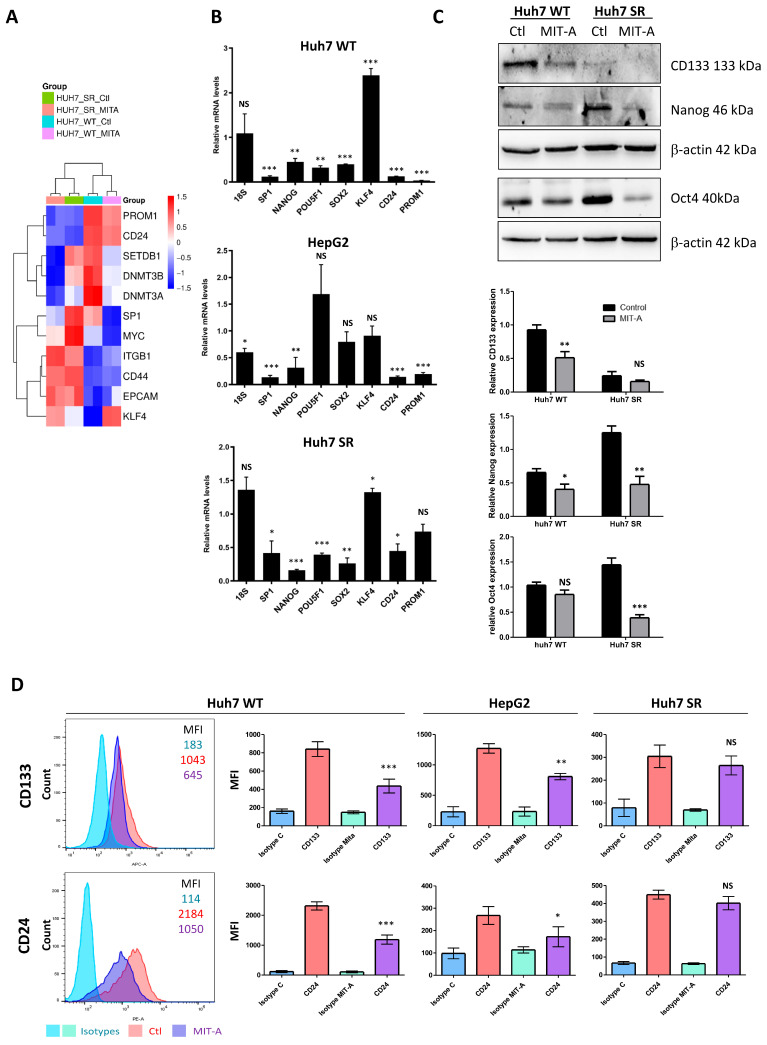
Effect of MIT-A on stemness-associated markers in HCC cells. (**A**) Heatmap showing the expression of stemness-associated genes (*PROM1*/CD133, *CD24*, *MYC*, *ITGB1*, *CD44*, *EPCAM*, *KLF4*) and direct SP1 targets (*SETDB1*, *DNMT3A*, *DNMT3B*) in Huh7 WT and SR cells after MIT-A treatment. (**B**) RT-qPCR analysis of SP1, pluripotency markers (*NANOG*, *SOX2*, *POU5F1*, *KLF4*), and surface markers (*CD24*, PROM1/*CD133*) in Huh7 WT, Huh7 SR, and HepG2 cells treated with MIT-A (200 nM, 24 h for Huh7 WT and HepG2; 400 nM, 24 h for Huh7 SR). Expression was normalized to 18S. (**C**) Western blot analysis of stemness regulators (OCT4, NANOG, CD133), SP1, and SETDB1 in Huh7 WT and SR cells treated with MIT-A. β-actin served as loading control. Representative blots and quantifications are shown. (**D**) Flow cytometry analysis of CSC surface markers (CD24, CD133) in Huh7 WT, Huh7 SR, and HepG2 cells after MIT-A exposure. Representative histograms are shown for Huh7 WT cells (red, control; violet, MIT-A; blue, isotype). Mean fluorescence intensity (MFI) values are shown on the right. Quantification is presented as bar graphs. Data are presented as mean ± SEM from independent experiments ((**B**,**C**): *n* = 3; (**D**): *n* = 4–6). Statistical analyses were performed using unpaired two-tailed Student’s *t*-tests (**B**) or Welch’s *t*-tests when appropriate (**C**,**D**). Statistical significance is indicated as * *p* < 0.05, ** *p* < 0.01, *** *p* < 0.001; NS, not significant.

We next evaluated the impact of MIT-A on stemness-associated markers in HCC cells. RNA-seq analysis of a curated panel of stemness-related genes (*PROM1*/CD133, *CD24*, *MYC*, *ITGB1*, *CD44*, *EPCAM*, *KLF4*) revealed that MIT-A treatment significantly reduced the mRNA levels of direct SP1 targets, including *SETDB1*, *DNMT3A*, and *DNMT3B*, with pronounced decreases in *PROM1*/CD133, *CD24*, and *MYC*, and an increase in *KLF4* in Huh7 WT cells. In Huh7 SR, MIT-A also reduced *MYC* expression and increased *KLF4* levels (Figure 4A). These findings were confirmed by quantitative PCR in Huh7 WT, HepG2, and Huh7 SR cells. Importantly, MIT-A downregulated the expression of key stemness-associated transcription factors (*OCT4*, *SOX2*, *NANOG*) and CSC surface markers (*PROM1*/CD133, *CD24*) in Huh7 WT cells (Figure 4B). Similar results were observed in Huh7 SR, except for *PROM1*/CD133 (not significant), as CD133 expression was low in this subline. Notably, *KLF4* expression increased following MIT-A treatment in both Huh7 WT and SR, suggesting an inverse regulatory effect. In contrast, HepG2 cells displayed a milder transcriptional response: *OCT4*, *SOX2*, *NANOG*, and *KLF4* showed a trend toward upregulation (not significant), while *PROM1*/CD133 and *CD24* were consistently reduced.

At the protein level, Western blot analyses corroborated these results, showing reduced CD133, OCT4 and NANOG protein levels in Huh7 WT and Huh7 SR following MIT-A treatment (Figure 4C). Flow cytometry confirmed that MIT-A significantly decreased the proportion of CD133^+^ and CD24^+^ cells in Huh7 WT and HepG2 (Figure 4D), whereas no significant changes were observed in Huh7 SR, consistent with their low baseline expression of these markers. Other stemness-related markers frequently reported in the literature, including EPCAM, CD29, CD105, CD44 and CD90, were largely unchanged, except for CD73, which showed increased surface expression upon MIT-A treatment (Supplementary Figure S3A). In HepG2 cells, the pattern was comparable, with consistent reductions in CD133 and CD24 and a modest decrease in CD105. Together, these results demonstrate that MIT-A consistently suppresses CSC transcriptional and protein programs across different genetic backgrounds and drug resistance states.

To mimic the tumor microenvironment, Huh7 WT and HepG2 cells were cultured under hypoxic conditions (1% O_2_). Hypoxia has been shown to induce SP1 expression, likely via HIF-1α-dependent mechanisms, enhancing the transcription of genes involved in cancer progression and stemness [27,28]. Consistently, we observed that hypoxia increased the expression of SP1, SETDB1, and stemness-associated genes (OCT4, SOX2, NANOG, CD133, CD24), promoting a CSC-like phenotype under low oxygen tension (Supplementary Figure S3B). Importantly, treatment with MIT-A abrogated this hypoxia-induced stemness phenotype. Flow cytometry analyses confirmed a significant reduction in CD133^+^ and CD24^+^ populations even in hypoxic cultures (Supplementary Figure S3C), indicating that pharmacological inhibition of SP1 effectively suppresses microenvironment-driven CSC programs.

Collectively, these findings demonstrate that MIT-A suppresses both basal and hypoxia-induced stemness programs in HCC cells, reducing transcription factors, CSC surface markers, and functional phenotypes in both parental and sorafenib-resistant settings.

### 3.5. MIT-A Reduces Functional CSC Properties in HCC Cells

**Figure 5:**
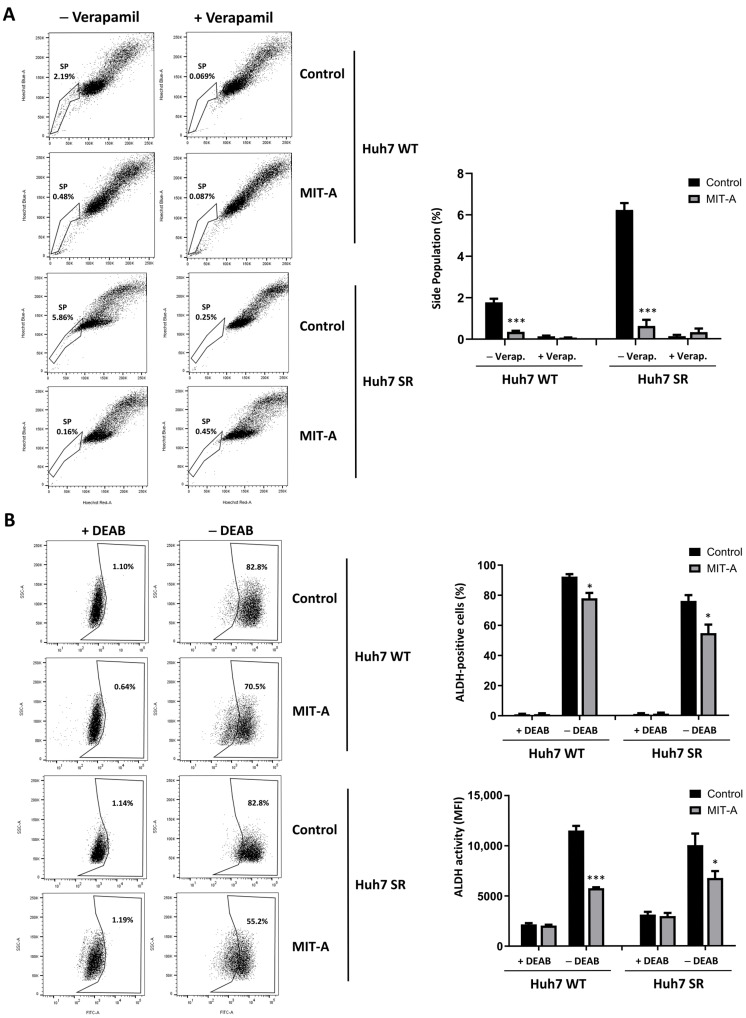
Effect of MIT-A on functional CSC properties. (**A**) Side population (SP) assay based on Hoechst 33,342 dye efflux in Huh7 WT and SR cells pretreated or not with MIT-A (200 nM, 24 h for Huh7 WT; 400 nM, 24 h for Huh7 SR). SP was analyzed by flow cytometry in the presence or absence of verapamil (50 μM). Representative SP plots are shown, and quantification of SP-positive cells is presented as bar graphs. (**B**) ALDH1A1 activity measured by Aldefluor assay in Huh7 WT and SR cells pretreated or not with MIT-A. DEAB (10 μM) was used as negative control. Right, representative histograms with percentages of ALDH-positive cells are shown. Representative histograms are shown, and quantification is presented as bar graphs indicating both the percentage of ALDH-positive cells and mean fluorescence intensity (MFI) as a measure of ALDH activity. Data are presented as mean ± SEM from independent experiments ((**A**,**B**): *n* = 4). Statistical analyses were performed using unpaired two-tailed Student’s *t*-tests or Welch’s *t*-tests when appropriate. Statistical significance is indicated as * *p* < 0.05, *** *p* < 0.001.

To determine whether MIT-A affects functional CSC properties beyond transcriptional and protein markers, we next investigated its impact on two established CSC-associated assays: the side population (SP) phenotype [25] and aldehyde dehydrogenase (ALDH) activity [29]. The SP phenotype identifies a subpopulation of cells with the ability to actively efflux DNA-binding fluorescent dyes such as Hoechst 33,342, a property mediated by ATP-binding cassette (ABC) transporters. This fraction is enriched in stem-like cells with self-renewal capacity and multipotency in both normal and malignant tissues [25]. Flow cytometry analysis showed that untreated Huh7 WT cells contained a small but detectable SP fraction (~2%), whereas Huh7 SR cells exhibited a three-fold higher baseline SP (~6%), consistent with their enhanced stem-like phenotype (Figure 5A). MIT-A treatment (200 nM, 24 h) markedly suppressed the SP fraction in both Huh7 WT and SR cells, indicating inhibition of CSC-associated efflux capacity.

High ALDH activity is another functional hallmark of CSCs in solid tumors, where it supports detoxification, resistance to oxidative stress, and differentiation plasticity [30,31]. Using the Aldefluor assay, we found that MIT-A treatment reduced both the frequency of ALDH-positive cells and the intensity of ALDH enzymatic activity (Figure 5B). In Huh7 WT cells, MIT-A slightly decreased the proportion of ALDH^+^ cells (*p* < 0.05) while significantly lowering their overall enzymatic activity (*p* < 0.05). In Huh7 SR cells, which displayed a higher basal ALDH activity than parental cells, MIT-A induced a more pronounced reduction (*p* < 0.001).

Together, these results demonstrate that MIT-A suppresses functional CSC properties in HCC cells, including dye efflux capacity and ALDH activity. These findings complement the transcriptional and protein-level data (Figure 4), confirming that inhibition of the SP1/SETDB1 axis suppresses both molecular and functional features of stemness in liver cancer.

### 3.6. MIT-A Sensitizes HCC Cells to Sorafenib

**Figure 6:**
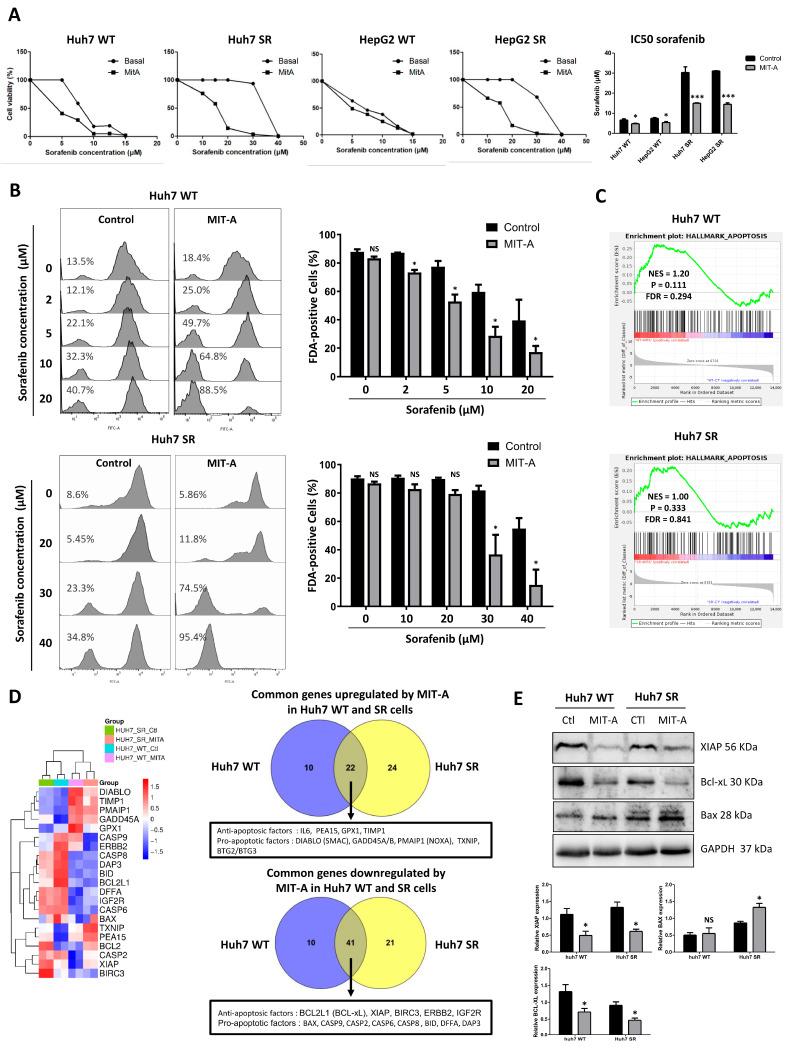
Effect of MIT-A on sorafenib sensitivity and apoptotic pathways. (**A**) Cell viability assays (CellTiter-Glo) in Huh7WT, HepG2WT, Huh7SR, and HepG2SR cells pretreated with MIT-A (200 nM for Huh7 WT and HepG2 WT; 400 nM for Huh7 SR and HepG2 SR, 24 h) and exposed to sorafenib (5–50 μM, 48 h). Viability curves are shown for each cell line, and IC_50_ values were determined by nonlinear regression. (**B**) Flow cytometry analysis of cell viability using FDA staining in Huh7 WT and SR cells exposed to sorafenib ± MIT-A. Representative histograms show FDA-negative (non-viable) cells, and quantification is presented as bar graphs. (**C**) GSEA of apoptosis-related Hallmark pathways in MIT-A–treated Huh7 WT and Huh7 SR cells. Representative enrichment plots are shown. NES, *p*-value, and FDR q-values are indicated. (**D**) RNA-seq analysis of apoptosis-related genes in Huh7 WT and Huh7 SR cells following MIT-A treatment. Left: heatmap showing modulation of pro- and anti-apoptotic genes. Right: Venn diagram showing the overlap of genes significantly modulated by MIT-A in Huh7 WT and Huh7 SR cells. (**E**) Western blot analysis of apoptosis-related proteins (XIAP, BCL-XL, BAX) in Huh7WT and Huh7SR cells treated with MIT-A. β-actin served as loading control. Representative blots and quantification graphs are shown. Bars represent mean ± SEM of *n* independent experiments (XIAP and BCL-xL WT, *n* = 5; SR, *n* = 3; Bax, *n* = 3). Statistical significance was determined using unpaired Welch’s *t*-test. Data are presented as mean ± SEM from independent experiments ((**A**): *n* = 3; (**B**): *n* = 4; (**E**): XIAP and BCL-XL WT, *n* = 5; SR, *n* = 3; BAX, *n* = 3). Statistical analyses were performed using Welch’s unpaired two-tailed *t*-tests for single comparisons (**A**,**E**) and two-way ANOVA followed by Sidak’s multiple comparisons test for viability assays (**B**). Statistical significance is indicated as * *p* < 0.05, *** *p* < 0.001; NS, not significant.

We next examined whether MIT-A modulates therapeutic response to sorafenib. Huh7 WT, HepG2 WT, and their sorafenib-resistant counterparts (Huh7 SR, HepG2 SR) were pre-treated with MIT-A (200 nM, 24 h) before exposure to increasing concentrations of sorafenib (5–20 µM for parental lines and 10–50 µM for SR lines). Cell viability was assessed using CellTiter-Glo assays (Figure 6A). Pre-treatment with MIT-A significantly enhanced sorafenib cytotoxicity in all tested cell lines, resulting in a significant reduction of IC50 values in both parental and sorafenib-resistant HCC cells. Notably, the sensitizing effect of MIT-A was particularly pronounced in resistant cells, where IC50 values were reduced by approximately 50%, indicating a robust restoration of drug sensitivity.

To ensure that the effect observed was not merely due to reduced proliferation (Figure 3 and Figure 4), we performed a complementary viability assay using fluorescein diacetate (FDA). FDA is converted by esterases of living cells into a fluorescent product, allowing discrimination between viable and dead cells by flow cytometry (Figure 6B). MIT-A pre-treatment enhanced sorafenib-induced cell death in a dose-dependent manner. In Huh7 WT, the percentage of FDA-positive living cells was significantly reduced in MIT-A–pretreated cultures starting at 5 µM sorafenib, with the difference becoming more pronounced at higher doses. Similar results were obtained in Huh7 SR cells, although the effect was observed at higher sorafenib concentrations (30–40 µM), consistent with their resistant phenotype.

Transcriptomic profiling and Gene Set Enrichment Analysis across 50 canonical Hallmark pathways showed that MIT-A treatment was significantly associated with the enrichment of apoptosis-related gene sets (Figure 6C). In Huh7 WT, MIT-A increased apoptosis hallmarks (NES = 1.20, *p* = 0.111), with a similar enrichment observed in Huh7 SR (NES = 1.00, *p* = 0.333), confirming that the drug reprograms apoptotic signaling in both parental and resistant settings.

At the gene level, MIT-A induced broad transcriptional changes in apoptosis regulators (Figure 6D). In both Huh7 WT and SR cells, several anti-apoptotic genes were upregulated, including *IL6*, *PEA15*, *GPX1*, and *TIMP1*, alongside pro-apoptotic mediators such as *DIABLO* (SMAC), *GADD45A/B*, *PMAIP1* (NOXA), and *TXNIP*. Conversely, a subset of anti-apoptotic genes (*BCL2L1*/BCL-XL, *XIAP*, *BIRC3*, *ERBB2*, *IGF2R*) as well as some pro-apoptotic factors (*BAX*, *CASP9*, *CASP2*, *CASP6*, *CASP8*, *BID*, *DFFA*, *DAP3*) were significantly downregulated by MIT-A. These results indicate a complex reprogramming of apoptotic pathways, converging toward increased susceptibility to sorafenib-induced death. Some transcriptomic changes were validated at the protein level by Western blotting (Figure 6E). In both Huh7 WT and SR, MIT-A reduced XIAP and BCL-XL expression while increasing BAX levels. Collectively, these data indicate that pre-treatment with MIT-A reprograms apoptosis regulators in a manner that enhances the sensitivity of both parental and sorafenib-resistant HCC cells to sorafenib-induced cell death.

Overall, these results demonstrate that MIT-A sensitizes both parental and sorafenib-resistant HCC cells to sorafenib. This chemosensitization is mediated, at least in part, through enhanced apoptosis and the suppression of key anti-apoptotic programs.

### 3.7. MIT-A Downregulates ABC Transporters and Reduces Efflux Activity

**Figure 7:**
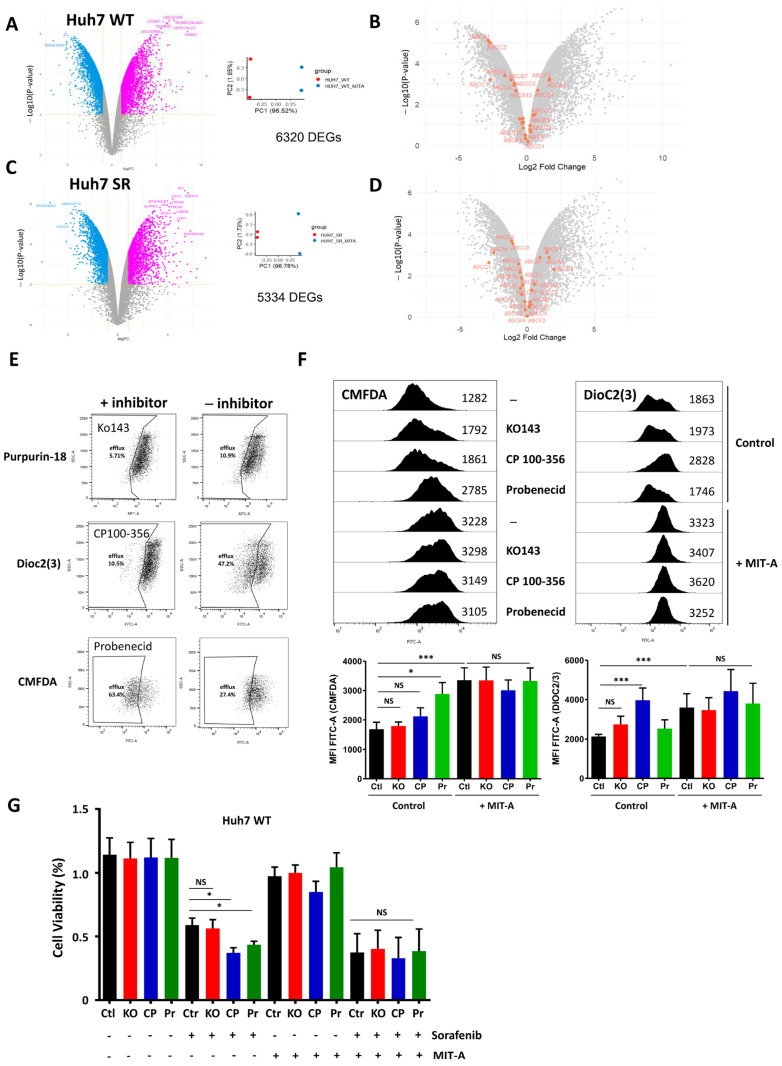
Effect of MIT-A on ABC transporter expression and efflux activity. (**A**,**C**) RNA-seq analysis of differentially expressed genes in Huh7 WT (**A**) and Huh7 SR (**C**) cells treated with MIT-A. Volcano plots display significantly up- and down-regulated genes. (**B**,**D**) RNA-seq analysis focusing on ABC transporter genes in Huh7 WT (**B**) and Huh7 SR (**D**) cells treated with MIT-A. Volcano plots display expression changes for individual transporters, including *ABCB1*, *ABCG2*, and members of the *ABCC* family. (**E**) Flow cytometry-based efflux assays in Huh7 WT cells using ABC transporter-specific fluorescent substrates (purpurin-18, DiOC2(3), and CMFDA) in the presence or absence of selective inhibitors (Ko143, CP100-356, and probenecid). After 30 min substrate incubation and washing, cells were resuspended in medium with or without inhibitors and incubated for 1 h at 37 °C. Efflux activity was assessed by flow cytometry. Representative histograms are shown with percentages of efflux-positive cells. (**F**) Flow cytometry analysis of dye accumulation in Huh7 WT cells pretreated or not with MIT-A. Cells were incubated with DIOC2(3) or CMFDA in the presence or absence of inhibitors. Representative histograms are shown with corresponding mean fluorescence intensity (MFI) values and quantification is presented as bar graphs. (**G**) Sorafenib cytotoxicity assays in Huh7 WT cells pretreated or not with MIT-A (200 nM) in the presence or absence of ABC transporter inhibitors (Ko143, CP100-356, or probenecid). Cells were exposed to sorafenib (10 μM, 48 h), and viability was measured using the CellTiter-Glo assay. Statistical significance was assessed using two-way ANOVA with MIT-A treatment and ABC transporter inhibition as independent variables, followed by Holm-Šídák post hoc tests for predefined comparisons. When appropriate, Welch’s unpaired *t*-tests were used for pairwise comparisons. Data are presented as mean ± SEM from independent experiments ((**F**,**G**): *n* = 4). Statistical analyses were performed using two-way ANOVA followed by Sidak’s multiple comparisons test for dye accumulation assays (**F**). For sorafenib cytotoxicity assays (**G**), statistical significance was assessed using two-way ANOVA with MIT-A treatment and ABC transporter inhibition as independent variables, followed by Holm–Šídák post hoc tests for predefined comparisons. When appropriate, Welch’s unpaired *t*-tests were used for pairwise comparisons. Statistical significance is indicated as * *p* < 0.05, *** *p* < 0.001; NS, not significant.

To further explore mechanisms underlying sorafenib sensitization, we investigated the effect of MIT-A on ABC drug efflux transporters in HCC cells. Transcriptomic profiling of Huh7 WT and Huh7 SR cells revealed that MIT-A significantly altered the expression of multiple ABC transporters. Differential expression analysis (Figure 7A,C) showed that several ABC transporter genes were downregulated following MIT-A treatment, including *ABCB1* (MDR1) and members of the *ABCC* family (*ABCC1–4*). Pathway-focused visualization (Figure 7B,D) highlighted that in both WT and SR cells, MIT-A consistently suppressed transporters associated with multidrug resistance, indicating a broad inhibitory effect on efflux-related transcriptional programs.

To determine whether these transcriptional changes translated into functional inhibition, we evaluated drug efflux activity using fluorescent substrates specific for ABC transporters (Figure 7E). Huh7 WT cells displayed minimal ABCG2 (BCRP) activity, with purpurin-18 efflux reduced from 11% to 6% following treatment with the selective ABCG2 inhibitor Ko143. In contrast, efflux of the ABCB1 (MDR1) substrate DIOC2(3) was strongly inhibited by the ABCB1-selective inhibitor CP100-356, decreasing from 47% to 10%, whereas efflux of the ABCC (MRP) substrate CMFDA was reduced by probenecid from 63% to 27%. These results confirm that Huh7 cells express functionally active ABCB1- and ABCC-mediated efflux systems, whereas ABCG2 activity is comparatively low.

We next assessed whether MIT-A directly impacts efflux activity (Figure 7F). In untreated Huh7 WT cells, CMFDA was efficiently extruded and specifically blocked by probenecid, confirming its dependence on ABCC activity. Upon MIT-A pretreatment, CMFDA accumulation increased in all conditions (with or without inhibitors), indicating that MIT-A reduced ABCC-associated efflux activity independently of pharmacological blockade. Similarly, DIOC2(3) was efficiently extruded and selectively inhibited by CP100-356 in untreated cells, confirming functional ABCB1 activity. In MIT-A–pretreated cells, DIOC2(3) accumulation increased across all conditions, consistent with a strong suppression of ABCB1-mediated efflux. Collectively, these findings indicate that MIT-A reduces ABCB1- and ABCC-associated efflux activity, overriding the effect of their specific pharmacological inhibitors.

To functionally link these observations to therapeutic response, we evaluated sorafenib cytotoxicity in Huh7 WT cells pretreated or not with MIT-A (200 nM, 24 h) in the presence or absence of ABC transporter inhibitors (Figure 7G). Under basal conditions without MIT-A, sorafenib reduced cell viability by approximately 50%. The addition of Ko143 (ABCG2 inhibitor) had no significant effect, consistent with the low ABCG2 activity observed. In contrast, CP100-356 (ABCB1 inhibitor) and probenecid (ABCC inhibitor) significantly enhanced sorafenib-induced cytotoxicity. Notably, in MIT-A-pretreated cells, sorafenib cytotoxicity was not further increased by the addition of transporter inhibitors, suggesting that MIT-A had already suppressed the contribution of ABCB1- and ABCC-mediated efflux to drug resistance. Thus, pharmacological inhibition of ABC transporters partially phenocopies the sensitizing effect of MIT-A, while MIT-A pretreatment largely overrides transporter-dependent resistance mechanisms.

Taken together, these findings indicate that MIT-A reduces ABC transporter expression and function, thereby limiting efflux activity and increasing cellular susceptibility to sorafenib. Although intracellular sorafenib levels were not directly measured in this study, the convergence between transporter inhibition and MIT-A pretreatment supports the interpretation that impaired efflux contributes to enhanced drug sensitivity. Future studies incorporating direct quantification of intracellular sorafenib will further clarify the extent to which transporter-dependent retention accounts for the observed therapeutic effect.

### 3.8. Genetic Silencing of SP1 Partially Recapitulates the Effects of Mithramycin A in HCC Cells

**Figure 8:**
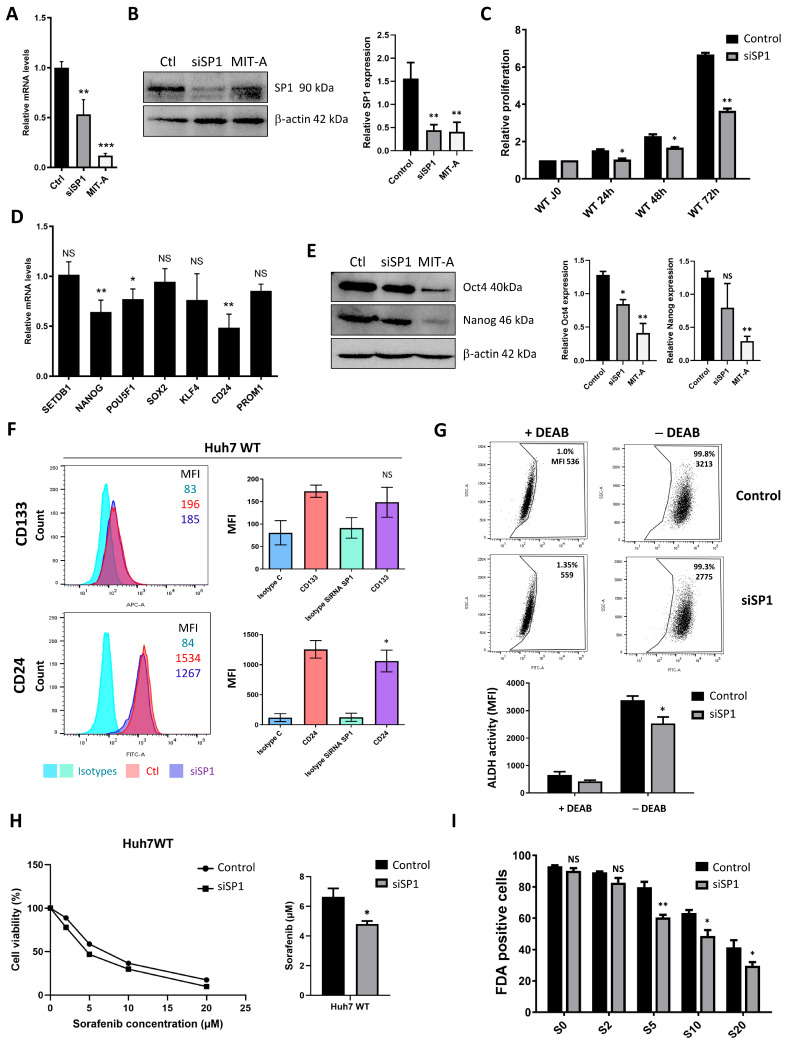
Genetic silencing of SP1 partially recapitulates the effects of pharmacological inhibition in HCC cells. (**A**) Validation of SP1 knockdown by RT-qPCR. Huh7 cells were transiently transfected with SP1-specific siRNA (siSP1) or control siRNA, with MIT-A (200 nM) included as a pharmacological control. SP1 mRNA expression was assessed 24 h post-transfection by RT-qPCR and normalized to 18S. Data are presented as mean ± SEM from at least three independent experiments. (**B**) Validation of SP1 protein downregulation by Western blot. SP1 protein levels were analyzed 48 h post-transfection by immunoblotting. β-actin served as loading control. Representative blots and densitometric quantification from three independent experiments are shown (mean ± SEM). (**C**) Effect of SP1 silencing on cell proliferation. Forty-eight hours after siRNA transfection, cell proliferation was monitored using the CellTiter-Glo luminescent assay and followed for 24–72 h. Luminescence values were normalized to the initial time point. Data represent mean ± SEM from at least three independent experiments. (**D**) Effect of SP1 silencing on stemness-associated transcription factors. Expression of OCT4 (POU5F1), NANOG, SOX2, SETDB1, PROM1/CD133, and KLF4 was evaluated by RT-qPCR 48 h post-transfection. Expression levels were normalized to 18S. Data are presented as mean ± SEM from at least three independent experiments. (**E**) Western blot validation of OCT4 and NANOG downregulation following SP1 silencing. Representative blots and densitometric quantification from three independent experiments are shown (mean ± SEM). (**F**) Flow cytometry analysis of CSC surface markers following SP1 knockdown. The proportion of CD24^+^ and CD133^+^ cells was quantified 48 h post-transfection. (**G**) Analysis of aldehyde dehydrogenase (ALDH) activity following SP1 silencing. The percentage of ALDH-positive cells and mean fluorescence intensity (MFI) were assessed by flow cytometry. (**H**,**I**) Effect of SP1 silencing on sorafenib sensitivity. Forty-eight hours after siRNA transfection, Huh7 cells were exposed to increasing concentrations of sorafenib (0, 2, 5, 10, and 20 µM). (**H**) Cell viability was assessed using the CellTiter-Glo assay to determine sorafenib IC_50_ values. (**I**) Viability was independently evaluated by fluorescein diacetate (FDA) staining followed by flow cytometry. Data are presented as mean ± SEM from independent experiments ((**A**–**F**,**H**): *n* = 3; (**G**): *n* = 3; (**I**): *n* = 4). For experiments involving multiple time points or drug concentrations (**C**,**H**,**I**), statistical analyses were performed using two-way ANOVA followed by Sidak’s multiple comparisons test. For comparisons between two groups (**A**,**B**,**D**–**G**), unpaired two-tailed Student’s *t*-test was used. Statistical significance is indicated as * *p* < 0.05, ** *p* < 0.01, *** *p* < 0.001; NS, not significant.

To genetically validate the contribution of SP1 to the phenotypes observed following MIT-A treatment, we performed transient SP1 silencing using specific siRNA in Huh7WT cells. Efficient knockdown of SP1 mRNA was confirmed by RT-qPCR 48 h post-transfection, demonstrating a reduction compared with control siRNA-transfected cells (Figure 8A). Consistently, Western blot analysis confirmed a decrease in SP1 protein levels under the same conditions (Figure 8B).

Functionally, SP1 silencing significantly reduced Huh7 cell proliferation, as assessed by CellTiter-Glo assay, indicating that SP1 contributes to the maintenance of proliferative capacity in HCC cells (Figure 8C). We next evaluated the impact of SP1 knockdown on stemness-associated transcription factors. RT-qPCR analyses revealed a marked decrease in *OCT4*, *NANOG* and *CD24* expression (Figure 8D). In contrast, no significant changes were observed for *SETDB1*, *SOX2*, *KLF4* or *PROM1*/CD133, under these conditions. Consistent with these transcriptional changes, Western blot analysis confirmed a significant reduction in OCT4 protein levels, while NANOG protein expression showed a decreasing trend that did not reach statistical significance (Figure 8E). To further evaluate CSC-associated phenotypes, we analyzed the expression of surface markers by flow cytometry. SP1 knockdown resulted in a reduction of CD24-positive cells, whereas no significant change was observed in the proportion of CD133-positive cells (Figure 8F). In addition, aldehyde dehydrogenase (ALDH) activity analysis revealed a decrease in ALDH fluorescence intensity (mean signal) in SP1-silenced cells, suggesting a partial reduction in ALDH activity (Figure 8G). Together, these findings indicate that SP1 silencing modulates a subset of stemness-associated transcriptional and phenotypic features, while other CSC markers appear to be regulated by additional mechanisms.

Given the previously observed enhancement of sorafenib sensitivity following MIT-A treatment, we next investigated whether SP1 silencing similarly modulates therapeutic response. SP1 knockdown significantly increased sorafenib-induced cytotoxicity in Huh7 cells, as demonstrated by both CellTiter-Glo and FDA-based viability assays (Figure 8H,I). Dose–response analyses revealed a shift in sorafenib sensitivity, with the calculated IC50 decreasing from 6.65 µM in control-transfected cells to 4.81 µM in SP1-silenced cells (Figure 8H). Consistently, FDA assays showed increased sorafenib-induced cell death at multiple concentrations (5, 10, and 20 µM) in SP1-silenced cells compared with control cells (Figure 8I). Although the magnitude of sensitization was less pronounced than that observed with MIT-A treatment, these results indicate that SP1 contributes to the regulation of sorafenib responsiveness in HCC cells.

Overall, genetic inhibition of SP1 partially phenocopied the anti-proliferative, anti-stemness, and sorafenib-sensitizing effects observed with MIT-A. Although the magnitude of these effects was less pronounced than with pharmacological inhibition, these findings support a significant contribution of SP1 to the regulation of stemness-associated programs and therapeutic resistance in HCC cells.

## 4. Discussion

HCC represents a global health challenge characterized by limited therapeutic options and poor patient outcomes. The restricted success of current therapies in HCC patients is largely attributed to tumor heterogeneity, which is dynamically driven by tumor-initiating stem cells, or CSCs. CSC subpopulations in HCC are characterized by distinct markers, including CD133, CD90, CD44, EpCAM, CD13, CD24, OV6, DLK1, α2δ1, ICAM-1, CD47, LGR5, and CK19 [4]. Among these, CD133 is particularly relevant, as its expression negatively correlates with overall survival and recurrence [32,33]. Patients with high CD133 expression display shorter 5-year survival and higher relapse after surgery [32], along with poor response to sorafenib [34]. CD133 upregulation also confers survival advantages under hypoxia or nutrient deprivation through autophagy activation [35]. Notably, double-positive CSC subsets, such as CD44^+^/CD90^+^ or CD44^+^/CD133^+^ cells, exhibit enhanced tumorigenicity and chemoresistance compared to single-positive populations, likely via stronger expression of stemness-related genes [36]. The remarkable plasticity of CSCs, driven by a range of genetic and epigenetic alterations, collectively confers resistance to classical therapeutic strategies [37]. Addressing this therapeutic challenge necessitates urgent development of personalized treatment strategies guided by predictive biomarkers, with particular emphasis on targeting CSC-associated regulatory networks.

In this context, transcriptional regulators such as SP1 have emerged as key integrators of oncogenic signaling and epigenetic programs that sustain proliferation, survival, and therapeutic resistance. SP1 controls gene expression both through direct promoter binding and indirectly via interactions with epigenetic enzymes (e.g., DNMT3A/3B, SETDB1, HDACs), chromatin remodelers, long non-coding RNAs, and RNA-binding proteins. Through this multilayered regulation, SP1 coordinates broad transcriptional networks linked to cell cycle progression, plasticity, and stress adaptation. In the present study, we combined TCGA-based transcriptomic analyses with pharmacological inhibition and complementary genetic silencing approaches to investigate the contribution of SP1-associated GC-rich transcriptional programs to CSC phenotypes and therapeutic resistance in HCC.

Consistent with this integrative role, SP1 expression was associated with patient prognosis in the TCGA-LIHC cohort. Importantly, SP1 overexpression appeared broadly conserved across major etiological HCC subtypes in the TCGA-LIHC cohort. High SP1 expression correlated with shorter disease-free survival and remained an independent prognostic factor in multivariable analysis, supporting its potential relevance as a biomarker of disease recurrence. However, the SP1 cutoff was determined using a maximally selected rank statistic and was not validated in an independent cohort. Therefore, these findings should be considered exploratory and require external validation. Future studies using continuous modeling of SP1 expression or cross-validated thresholds may provide more robust estimates of its prognostic value.

To further investigate the functional and mechanistic basis underlying these clinical and transcriptomic observations, we next examined the effects of pharmacological inhibition of GC-rich transcriptional programs using mithramycin A (MIT-A) in HCC cell models. Our study showed that MIT-A treatment reduced SP1 protein levels across multiple HCC cell lines in vitro and was associated with transcriptome-wide suppression of proliferation-related gene programs, including E2F targets, G2M checkpoint regulators, and mitotic spindle components. These transcriptional alterations were consistent with the observed reduction in cell proliferation following MIT-A exposure, supporting a role for GC-rich transcriptional regulation in sustaining cell cycle progression in HCC. Complementary genetic silencing experiments further supported a contributory role for SP1 in growth control. SP1 knockdown reduced proliferation, although the magnitude of the effect was less pronounced than that observed with MIT-A treatment. This difference indicates that MIT-A does not act solely through SP1 inhibition, but more broadly interferes with GC-rich transcriptional programs involving multiple transcription factors. Together, these findings indicate that SP1-associated transcriptional programs participate in the maintenance of proliferative capacity in HCC cells.

Beyond its impact on proliferation, MIT-A treatment markedly suppressed migratory capacity in scratch-wound assays, particularly in Huh7 SR cells, which exhibited higher basal motility than parental Huh7 WT cells. This reduction in migration was not associated with a consistent or coordinated EMT reversion pattern, suggesting that alternative mechanisms underlie this phenotype. Transcriptomic analyses revealed coordinated downregulation of genes involved in cytoskeletal organization and actin remodeling, including regulators of Rho GTPase signaling and components of the actin polymerization machinery. These findings were supported by immunofluorescence data demonstrating reduced pseudopodia formation and loss of actin-based protrusions following MIT-A treatment. Together, these observations indicate that GC-rich transcriptional programs contribute to cytoskeleton-driven migratory behavior in HCC cells. Migration was not directly evaluated following SP1 silencing in our experimental setting; therefore, we cannot determine the extent to which this phenotype is exclusively SP1-dependent. The more pronounced effect observed with MIT-A suggests that broader GC-rich transcriptional interference may contribute to suppression of migratory behavior beyond isolated SP1 depletion. Nevertheless, previous reports have linked SP1 to invasive phenotypes in liver and other cancers [17,38,39]. For example, miR-612, which suppresses SP1 expression [21], also regulates invadopodia formation in hepatocellular carcinoma through HADHA-mediated lipid reprogramming [40]. Although our study did not directly assess SP1 occupancy at specific cytoskeletal promoters, these observations are consistent with a contributory role for SP1-associated transcriptional networks in sustaining invasive cellular programs.

SP1-associated transcriptional programs may also intersect with receptor-driven invasive signaling networks. The HGF/c-MET axis is a recognized regulator of invasion and metastasis in HCC. In our RNA-seq analyses, MIT-A treatment was associated with partial attenuation of c-MET-related gene signatures, suggesting that GC-rich transcriptional regulation may contribute to reinforcement of pro-invasive signaling outputs downstream of receptor activation. We did not directly assess MET phosphorylation or receptor activity; therefore, our data do not demonstrate direct SP1 regulation of c-MET signaling. Rather, these findings are consistent with a model in which SP1 functions as a transcriptional amplifier of invasive gene programs that overlap with c-MET–associated pathways. From a translational perspective, c-MET has been investigated as a therapeutic target in advanced HCC, notably through the evaluation of tivantinib. Although early studies suggested benefit in selected subgroups, subsequent trials failed to demonstrate consistent survival advantage and raised safety concerns [41]. These outcomes underscore the redundancy and adaptability of oncogenic signaling in HCC and suggest that modulation of upstream transcriptional regulators may provide an alternative strategy to indirectly influence multiple pro-invasive signaling outputs without relying solely on receptor blockade.

In addition to its role in proliferation and migration, SP1 was strongly associated with stemness regulators and stemness signatures in HCC. TCGA analyses revealed positive correlations between SP1 expression and pluripotency-associated transcription factors, including OCT4, NANOG, and SOX2. Pharmacological inhibition with MIT-A attenuated these factors at both transcript and protein levels under normoxic conditions and reduced CSC surface markers such as CD133 and CD24. Beyond transcriptional changes, MIT-A impaired functional CSC properties, including Side Population frequency and ALDH activity in various HCC cell lines, indicating that GC-rich transcriptional interference affects CSC-enriched phenotypes at both molecular and functional levels. Notably, MIT-A also attenuated hypoxia-induced enrichment of CSC markers, as hypoxic culture conditions increased CD133 and CD24 expression whereas MIT-A treatment abrogated this response (Supplementary Figure S3). This observation is consistent with the well-established role of hypoxia in reinforcing CSC plasticity in HCC through HIF-dependent transcriptional programs and adaptive stress responses [35].

In this context, our findings suggest that SP1 contributes to the maintenance of CSC subpopulations in HCC. SP1’s role in regulating cancer stemness has been supported by studies using mithramycin A, siRNA-mediated depletion, and SP1 overexpression models. In colorectal and glioma systems, SP1 has been reported to regulate PROM1/CD133 expression and associated stem-like properties via GC-rich promoter elements [14,15]. In lung cancer, SP1 and SP3 regulate ABCG2 (BCRP) expression to sustain the side population phenotype and chemoresistance [16]. Collectively, these findings support a role for SP family transcription factors in CSC-associated transcriptional circuitry across tumor types, reinforcing the biological plausibility of SP1 involvement in HCC stemness. Complementary SP1 silencing experiments in our study partially supported these observations. SP1 knockdown reduced the expression of key pluripotency-associated transcription factors, notably OCT4 and NANOG, consistent with previous reports in HCC showing that miR-612 suppresses stem cell-like traits by targeting SP1 and its downstream effector NANOG [21]. In addition, a modest decrease in CD24 surface expression and ALDH activity was observed following SP1 depletion, further suggesting a partial attenuation of CSC-associated phenotypes. However, not all stemness markers were affected under these conditions, as *PROM1*/CD133 and KLF4 expression remained largely unchanged. Together, these findings indicate that SP1 contributes to the regulation of a subset of stemness-associated transcriptional programs in HCC cells, while additional regulatory mechanisms likely control other CSC markers. In this context, the broader effects observed following MIT-A treatment may reflect interference with GC-rich promoter occupancy and the activity of additional GC-binding transcription factors in our experimental system.

Importantly, the magnitude of stemness attenuation achieved with SP1 silencing was less pronounced than that observed following MIT-A treatment. Several mechanisms may account for this difference. Functional redundancy within the SP family likely compensates for isolated SP1 depletion, as SP1, SP3 and SP4 recognize identical GC-box motifs through highly conserved zinc-finger domains [42]. SP3 can occupy GC elements and function as an activator or repressor depending on promoter context, thereby buffering the effect of SP1 loss [43]. Consistently, combined SP1/SP3 depletion produces stronger transcriptional repression than single knockdown, approaching the effects of mithramycin A [16]. Similar cooperative requirements have been reported in other systems: McDermott et al. showed that simultaneous SP1 and SP3 knockdown was necessary to repress GPER1 expression [44], whereas individual inhibition had minimal impact, and Meinders et al. demonstrated that double genetic ablation of SP1 and SP3 resulted in severe thrombocytopenia, while single loss produced only modest phenotypes, underscoring their functional synergy [45]. In addition, mithramycin A chemically saturates GC-rich DNA sequences, preventing binding of multiple GC-box-recognizing transcription factors simultaneously, which may generate broader transcriptional inhibition than partial (~70–80%) reduction of a single protein by siRNA. Residual SP1 protein, due to its relatively long half-life, may further maintain promoter occupancy after knockdown. Together, these considerations provide a mechanistic explanation for the partial effects observed with SP1 silencing compared with the broader transcriptional interference induced by MIT-A.

To further explore the molecular basis underlying the differential effects observed between pharmacological inhibition and SP1 silencing, we examined the expression of key epigenetic regulators associated with stemness. The attenuation of stemness-associated phenotypes following MIT-A treatment was accompanied by reduced expression of epigenetic effectors, including SETDB1, DNMT3A, and DNMT3B. SETDB1, a histone H3K9 methyltransferase, has been identified as a hallmark of aggressive, stemness-enriched HCC subtypes and contributes to transcriptional repression programs linked to tumor progression [46,47]. Direct transcriptional regulation of SETDB1 by SP family members has been demonstrated in other contexts. In neuronal models, SP1 and SP3 activate the ESET/SETDB1 promoter, and mithramycin interferes with their promoter occupancy [48]. In gastric cancer, SP1 and β-catenin bind the SETDB1 promoter and regulate tumor progression through a SETDB1-dependent axis [49]. In our HCC models, MIT-A reduced SETDB1 expression at both transcript and protein levels. However, SP1 silencing did not significantly alter SETDB1 expression under our experimental conditions. This discrepancy suggests that SETDB1 regulation in HCC may involve cooperative or compensatory GC-binding transcription factors, including SP3 or other GC-box-associated regulators, rather than exclusive SP1 dependency. Accordingly, MIT-A-mediated SETDB1 downregulation likely reflects broader interference with GC-rich promoter occupancy rather than isolated SP1 depletion. Similarly, DNMT3A and DNMT3B have been implicated in CSC maintenance and therapy resistance. DNMT3A can repress Hippo pathway regulators via MORC2 [50], and IL-6/STAT3-driven DNMT3B upregulates OCT4, reinforcing CSC traits and resistance to sorafenib [51]. In our study, MIT-A reduced DNMT3A and DNMT3B expression, supporting the involvement of GC-rich transcriptional programs in epigenetic reinforcement of stemness-associated networks. Collectively, these findings indicate that SP1-associated GC-rich transcriptional circuits intersect with epigenetic regulators that sustain CSC phenotypes in HCC, while also underscoring that pharmacological interference extends beyond isolated SP1 depletion.

Sorafenib is a multi-kinase inhibitor that has long represented a cornerstone in the systemic treatment of advanced HCC, being the first agent to demonstrate a survival benefit in Phase III trials for unresectable disease [52,53]. However, resistance frequently emerges through adaptive mechanisms, including activation of survival signaling pathways, enrichment of CSC-associated programs, and increased drug efflux mediated by ABC transporters [1,2].

In the current era of precision medicine, the identification of biomarkers associated with therapeutic resistance represents a major unmet need in HCC management. Despite the widespread clinical use of sorafenib, robust predictive biomarkers of treatment response remain lacking. Interestingly, several studies have reported associations between sorafenib-related adverse events, particularly dermatological toxicities, and improved therapeutic efficacy, although these correlations remain indirect and inconsistently validated across patient cohorts [54]. These observations underscore the urgent need for molecular biomarkers capable of improving patient stratification and guiding treatment selection in advanced HCC. In this context, characterization of transcriptional programs associated with CSC maintenance and drug resistance may help identify biologically distinct subgroups of HCC with differential sensitivity to systemic therapies. Importantly, improved molecular stratification of sorafenib-resistant tumors could also facilitate the identification of patients potentially responsive to alternative therapeutic strategies following sorafenib failure, including metronomic capecitabine-based approaches [55].

In line with this clinical need, our findings show that pharmacological inhibition of GC-rich transcriptional programs with MIT-A sensitizes HCC cells to sorafenib in both parental cell lines and experimentally derived sorafenib-resistant variants. Complementary SP1 silencing in Huh7 WT cells modestly enhanced sorafenib-induced cytotoxicity, supporting a contributory role for SP1 in TKI response while indicating that pharmacological interference may exert broader transcriptional effects. Two dominant mechanisms emerged from our study as central contributors to MIT-A-mediated sensitization to sorafenib. First, MIT-A pretreatment shifted the apoptotic balance in favor of cell death. Mechanistically, RNA-seq and immunoblotting revealed concomitant downregulation of anti-apoptotic mediators (Bcl-XL, XIAP) and upregulation of pro-apoptotic factors (Bax). This rebalancing is consistent with SP1’s known transcriptional regulation of apoptosis genes [8] and with prior reports demonstrating that mithramycin can downregulate XIAP and Bcl-2 and sensitize tumor cells to genotoxic drugs [56,57]. In this context, SP1 emerges as an upstream coordinator of survival networks that condition TKI response [7]. Moreover, our findings align with evidence that SP1-centered circuits, such as the LINC01134/SP1/p62 axis, reinforce oxidative stress tolerance and chemotherapy resistance [58]. Importantly, these results also resonate with previous findings showing that CD133-positive hepatic CSCs exhibit greater chemoresistance than their CD133-negative counterparts through activation of pro-survival Akt/PKB and anti-apoptotic Bcl-2 signaling cascades [59].

Second, MIT-A attenuated drug efflux capacity by repressing ABC transporters [57]. ABC family members, including ABCB1 (MDR1), ABCG2 (BCRP), and several ABCC transporters (MRPs), are well-established mediators of multidrug resistance in HCC [60]. Our RNA-seq analyses and functional experiments showed that MIT-A reduced transporter activity, most notably affecting ABCB1- and ABCC-mediated efflux, whereas ABCG2 activity was not significantly altered. Pharmacological inhibition of ABC transporters partially mimicked the sensitizing effect of MIT-A, and MIT-A pretreatment reduced the additional benefit conferred by pump blockade. Although intracellular sorafenib accumulation was not directly quantified, these findings are consistent with reduced efflux capacity contributing to enhanced drug cytotoxicity. Together, these results indicate that MIT-A-mediated interference with GC-rich transcriptional networks enhances sorafenib response through complementary effects on apoptotic priming and efflux restriction in both naïve and resistant HCC cells.

From a translational perspective, further validation in physiologically relevant models will be required before clinical application of GC-rich transcriptional targeting strategies in HCC. In particular, patient-derived organoids, xenograft models, and orthotopic in vivo systems may help clarify the impact of SP1-associated transcriptional programs on tumor heterogeneity, CSC plasticity, and therapeutic response in complex tumor microenvironments [4]. Moreover, although mithramycin A demonstrated promising anti-tumoral activity in our experimental models, its historical clinical development has been limited by systemic toxicities, particularly hepatotoxicity. In this context, the emergence of next-generation mithramycin analogs with improved pharmacological properties, such as EC-8042, may provide new opportunities for translational development of GC-rich transcriptional targeting approaches in liver cancer [61].

Several limitations warrant consideration. Although SP1 silencing supports a contributory role for SP1 in sorafenib response, the broader pharmacological profile of MIT-A likely involves additional GC-binding transcription factors. Chromatin occupancy studies would help clarify the relative contribution of SP1 versus other SP family members. Moreover, although our in vitro models included both parental and sorafenib-resistant cell lines, additional validation in patient-derived and in vivo systems will be necessary to further confirm translational applicability. Finally, direct quantification of intracellular sorafenib concentrations would further strengthen the mechanistic link between reduced efflux and enhanced drug sensitivity.

In conclusion, this work supports a role for SP1-associated GC-rich transcriptional programs in regulating stemness-associated phenotypes and therapeutic resistance in HCC. Pharmacological disruption of these programs with MIT-A attenuated proliferation and migration, reduced CSC-associated features, and enhanced sorafenib cytotoxicity.

Mechanistically, our findings suggest that MIT-A acts through broad interference with GC-rich transcriptional networks extending beyond isolated SP1 inhibition. These effects appear to involve coordinated modulation of multiple resistance-associated mechanisms, including attenuation of stemness-associated transcriptional programs, rebalancing of apoptotic signaling, repression of ABC transporter-mediated efflux, and interference with cytoskeleton-related migratory pathways. Collectively, these findings provide a rationale for exploring transcriptional-epigenetic modulation strategies targeting GC-rich regulatory networks in combination with standard therapies. Although the clinical use of MIT-A has historically been limited by hepatotoxicity [62], next-generation mithramycin analogs such as EC-8042 have been developed to improve pharmacological properties and reduce systemic toxicity [61]. Future studies should prioritize in vivo validation, mechanistic dissection in patient-derived systems, and identification of predictive biomarkers to refine patient stratification.

## Data Availability

The RNA-seq data presented in this study are openly available in FigShare at https://doi.org/10.6084/m9.figshare.31979637 (accessed on 10 April 2026), reference number 31979637.

## Supplementary Materials

The following supporting information can be downloaded at: https://www.mdpi.com/article/10.3390/cells15110961/s1, Figure S1: SP1 expression according to hepatocellular carcinoma etiological subtypes in the TCGA-LIHC cohort; Figure S2: Characterization of sorafenib-resistant cells and effect of MIT-A on cytoskeletal organization; Figure S3: Hypoxia induction of stemness and effect of MIT-A; Table S1: Clinicopathological characteristics of the TCGA-LIHC cohort included in the study; Table S2: Primers and Probes Used for qRT-PCR.
