# Supplementary material for: Pharmacological Inhibition of SP1 Reverses Cancer Stemness and Enhances Sorafenib Efficacy in Hepatocellular Carcinoma
Source: Cells. 2026 May 22;15(11):961. doi: 10.3390/cells15110961 (PMC13256930; doi:10.3390/cells15110961)
Supplement: Supplementary file 1 [file cells-15-00961-s001.zip › cells-4289091-supplementary.pdf]

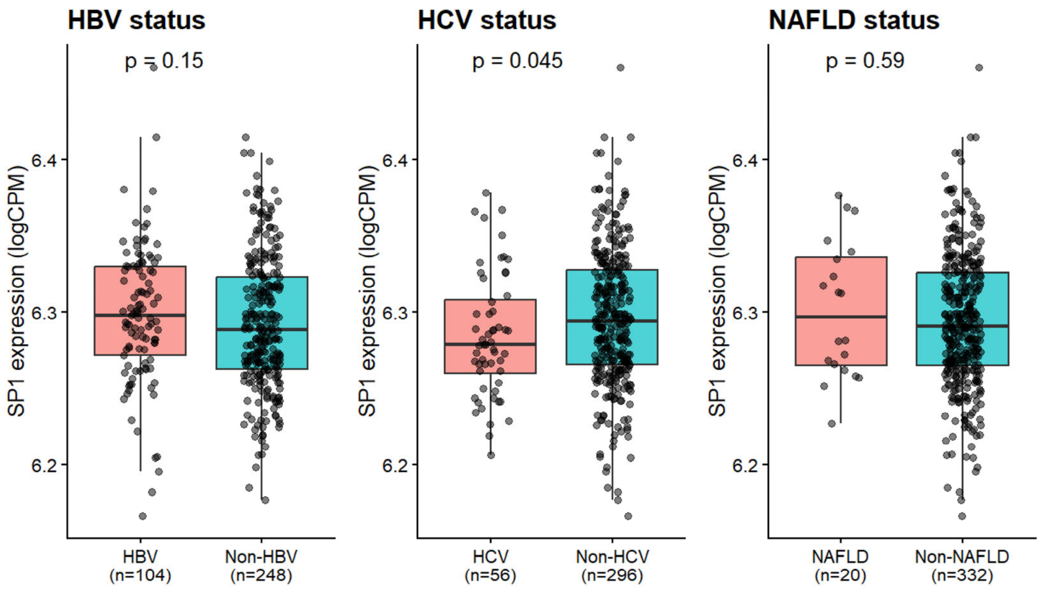

Figure S1. SP1 expression according to hepatocellular carcinoma etiological subtypes in the TCGA-LIHC cohort. Boxplots showing SP1 mRNA expression levels in HCC tumors stratified according to (A) HBV status, (B) HCV status, and (C) NAFLD status in the TCGA-LIHC cohort. SP1 expression was broadly comparable across etiological subgroups. Although a marginal statistical difference was observed between HCV-associated and non-HCV tumors, the magnitude of variation remained minimal. Statistical analyses were performed using two-sided Wilcoxon rank-sum tests.

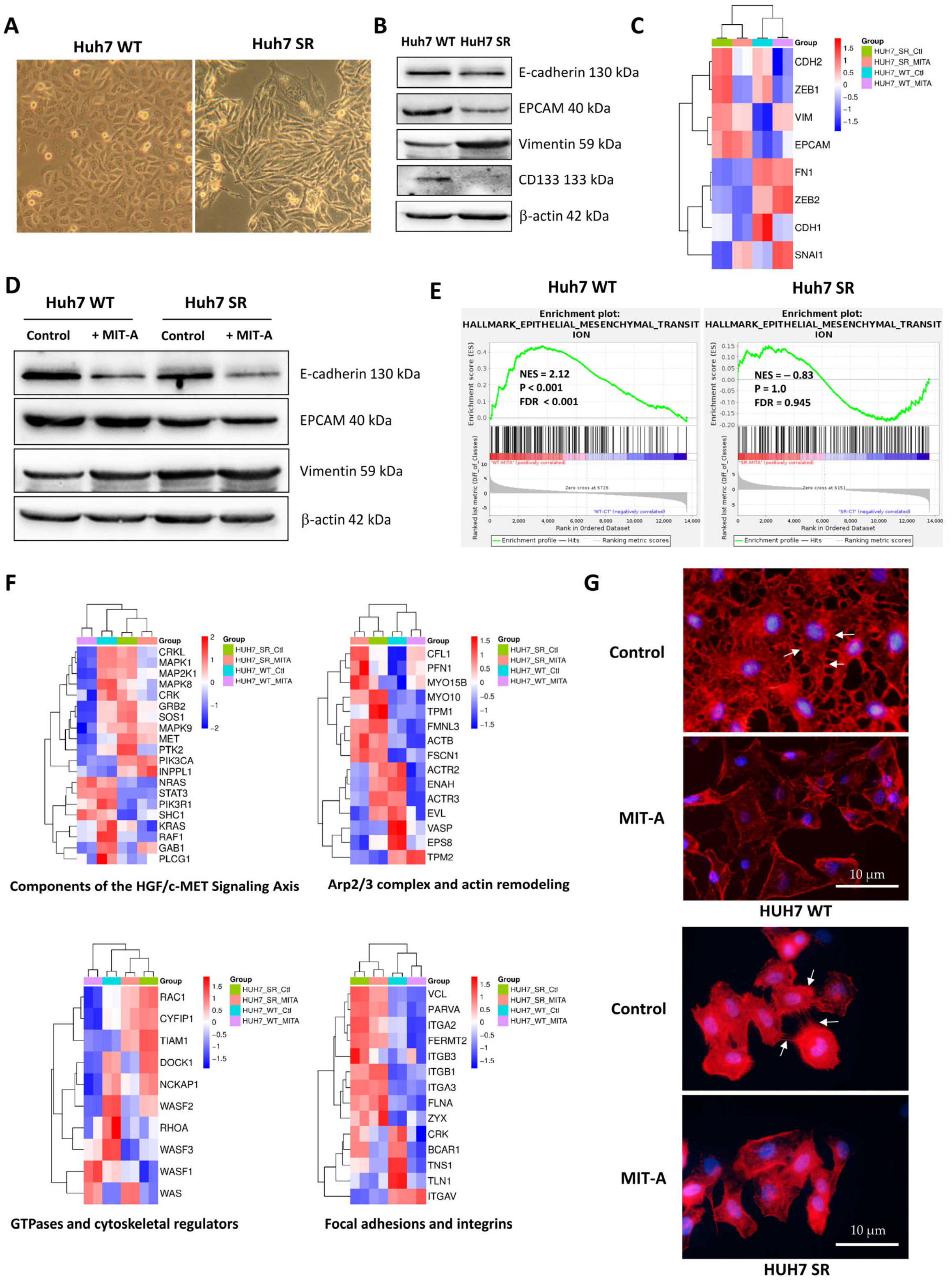

Figure S2. Characterization of sorafenib-resistant cells and effect of MIT-A on cytoskeletal organization. (A) Morphological and molecular characterization of Huh7 SR compared with Huh7 WT cells. Representative phase-contrast images. (B) Western blot analysis of EMT markers (E-cadherin, EpCAM, Vimentin) and CD133 in Huh7 WT and SR cells. β-actin served as loading control. (C) Heatmap showing the expression of EMT-related genes (CDH1, CDH2, VIM, EPCAM, SNAI1, ZEB1, ZEB2) in Huh7 WT and SR cells after MIT-A treatment. Color scale

indicates normalized expression values (log2 TPM or z-score). (D) Western blot analysis of EMT markers in Huh7 WT and SR cells treated with MIT-A. β-actin served as loading control. (E) GSEA of epithelial–mesenchymal transition-related Hallmark pathways in MIT-A–treated Huh7 WT and SR cells. NES, p-value, and FDR q-values are indicated. (F) RNA-seq analysis of migration regulators in MIT-A–treated HCC cells, including HGF/c-MET signaling axis, Rho GTPases, the Arp2/3 actin remodeling complex, and integrin/focal adhesion components. (G) Immunofluorescence analysis of F-actin pseudopodia in Huh7 WT and SR cells treated or not with MIT-A (200 nM, 48 h). Representative images show phalloidin (green or red) and DAPI (blue). White arrowheads indicate pseudopodia. Scale bar = 10 μm. Each experiment was performed three times.

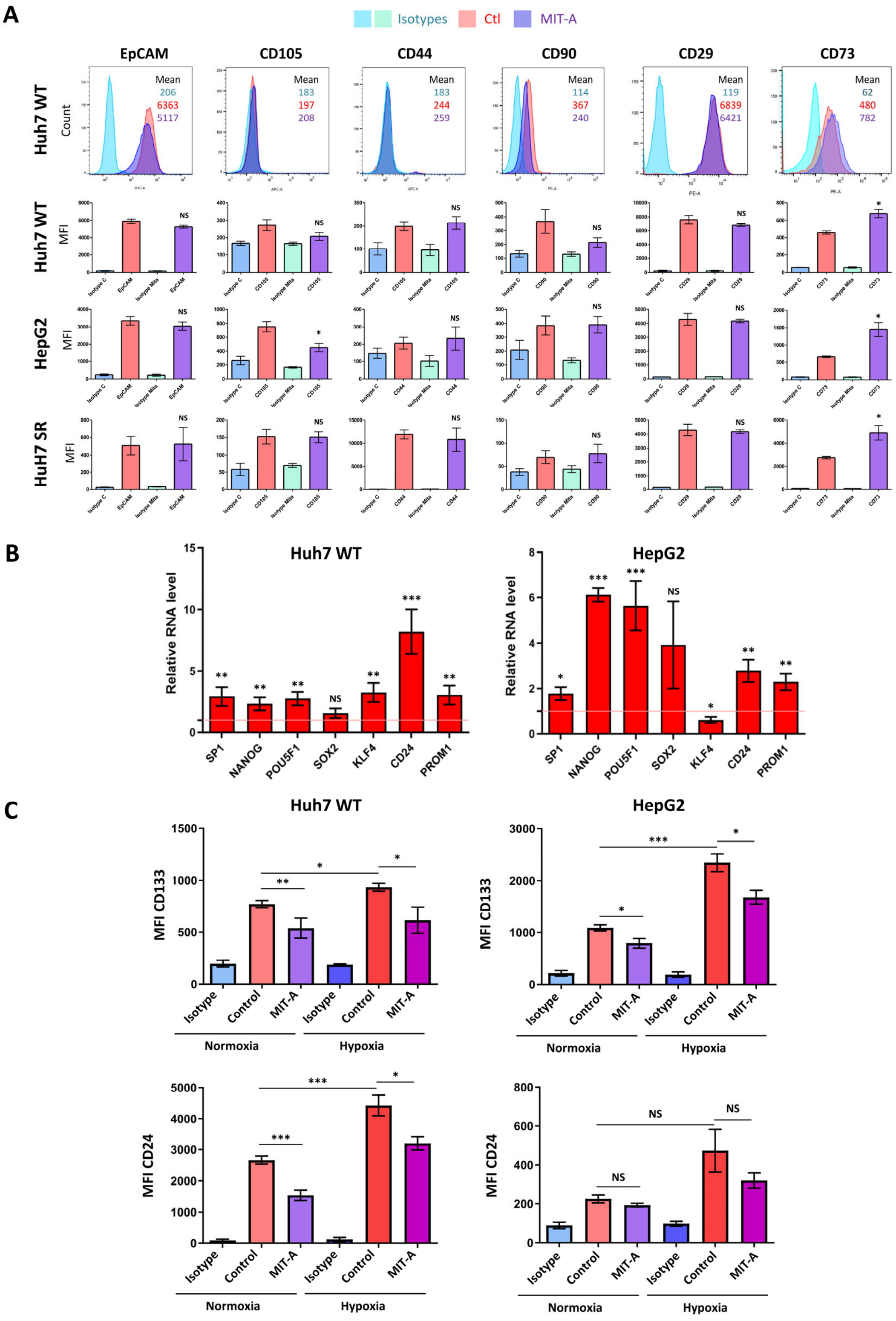

Figure S3. Hypoxia induction of stemness and effect of MIT-A. (A) Flow cytometry analysis of surface markers (CD29, CD44, CD73, CD90, CD105, EpCAM) in Huh7 WT, Huh7 SR, and HepG2 cells after MIT-A treatment (200 nM, 48 h for Huh7 WT and HepG2; 400 nM, 48 h for Huh7 SR). Representative histograms are shown for Huh7 WT cells (red, control; violet, MIT-A; blue, isotype). MFI values are indicated; quantification is shown as bar graphs (mean ± SEM, n = 3-4). (B) RT-qPCR analysis of SP1 and SP1 targets (*SETDB1*, *DNMT3A*, *DNMT3B*), pluripotency factors (*NANOG*, *SOX2*, *POU5F1*, *KLF4*),

and surface markers (*CD24*, *PROM1*/CD133) in Huh7 WT and HepG2 cells cultured under hypoxia (1% O_2). Expression was normalized to 18S. Data are mean ± SEM (n = 3). (C) Flow cytometry analysis of CD133 and CD24 in Huh7 WT and HepG2 cells under normoxia, hypoxia, with or without MIT-A (200 nM, 48 h). Representative histograms are shown for Huh7 WT cells: staining with specific antibodies (red for normoxia, grey for hypoxia, violet for hypoxia + MIT-A) and isotype controls (blue). Quantification of MFI values is presented as bar graphs (mean ± SEM; n = 3-4).

Table S1. Clinicopathological characteristics of the TCGA-LIHC cohort included in the study.

Variable	Level	TCGA liver (n=371)
gender	Male	250 (67.4)
	Female	121 (32.6)
AGE	mean (sd)	59.4 (13.5)
	missing	1
NAFLD	no	332 (94.3)
	YES	20 (5.7)
	missing	19
Hepatitis_B	no	248 (70.5)
	YES	104 (29.5)
	missing	19
Hepatitis_C	no	296 (84.1)
	YES	56 (15.9)
	missing	19
Alcohol_consumption	no	235 (66.8)
	YES	117 (33.2)
	missing	19
Tobacco	YES	15 (48.4)
	no	16 (51.6)
	missing	340
VASCULAR_INVASION	Macro	16 (5.1)
	None	206 (65.4)
	Micro	93 (29.5)
	missing	56
AFP_AT_PROCUREMENT	mean (sd)	13,987.2 (125,463.8)
	missing	93
CHILD_PUGH_CLASSIFICATION	A	217 (90.8)
	B	21 (8.8)
	C	1 (0.4)
	missing	132
GRADE	G3	122 (33.3)
	G2	177 (48.4)
	G1	55 (15.0)
	G4	12 (3.3)
	missing	5

Table S2. Primers and Probes Used for qRT-PCR.

Target Gene Symbol	Gene Name	Tamqan Assay ID (ThermoFisher)	Probe Chemistry
SP1	Specific Prtotein 1	Hs00916521_m1	FAM-MGB
SETDB1	SET domain bifurcated 1	Hs01048361_m1	FAM-MGB
PROM1	Prominin 1	Hs01009259_m1	FAM-MGB
CD24	CD24 molecule	Hs02379687_s1	FAM-MGB
POU5F1	POU class 5 homeobox 1	Hs03005111_g1	FAM-MGB
KLF4	Kruppel like factor 4	Hs00358836_m1	FAM-MGB
NANOG	Nanog homeobox	Hs02387400_g1	FAM-MGB
SOX2	SRY-box 2	Hs04234836_s1	FAM-MGB
18S	Eukaryotic 18S rRNA	Hs03003631_g1	FAM-MGB
